# Muscle-Specific DNM2 Overexpression Improves Charcot–Marie–Tooth Disease In Vivo and Reveals a Narrow Therapeutic Window in Skeletal Muscle

**DOI:** 10.3390/ijms27031471

**Published:** 2026-02-02

**Authors:** Marie Goret, Gwenaelle Piccolo, Jocelyn Laporte

**Affiliations:** Institute of Genetics and Molecular and Cellular Biology (IGBMC), INSERM U1258, CNRS UMR7104, University of Strasbourg, 67404 Illkirch, France

**Keywords:** Hereditary motor and sensory neuropathy, HMSN, Charcot–Marie–Tooth neuropathy, CMT, dynamin, GTPase, adeno-associated virus, AAV, gene therapy, myopathy, muscle

## Abstract

Charcot–Marie–Tooth disease (CMT), caused by dominant loss-of-function mutations in *DNM2*, encoding the GTPase dynamin-2, impairs motor and sensory function. However, the respective contributions of muscle and nerve pathology, and the therapeutic potential of increasing DNM2 expression, remain unresolved. We evaluated tissue-targeted and systemic approaches to increase DNM2 in a mouse model carrying the common K562E-CMT mutation. Muscle-specific DNM2 overexpression from embryogenesis in *Dnm2^K562E/+^* mice ameliorated desmin and integrin mislocalization, membrane trafficking defects, mitochondrial abnormalities, and fibrosis in skeletal muscle, resulting in improved locomotor coordination despite persistent muscle atrophy. Conversely, systemic postnatal AAV delivery of human DNM2 increased DNM2 in muscle but failed to transduce nerves and paradoxically worsened the muscle pathology, producing centronuclear myopathy-like features. These findings reveal a primary pathogenic impact of *DNM2*-CMT mutation within skeletal muscle, independent of nerve involvement. Collectively, they underscore that precise DNM2 dosage is critical for neuromuscular homeostasis and reveal a narrow therapeutic window for safe and effective therapeutic intervention. This paradox, in which efforts to compensate for a loss-of-function neuropathy risk inducing a gain-of-function myopathy, highlights the need for tightly controlled modulation of DNM2 activity in future therapeutic strategies.

## 1. Introduction

Charcot–Marie–Tooth (CMT) disease, one of the most common inherited peripheral neuropathies (prevalence ~1:2500), arises from mutations in over 100 genes [1]. Among these, heterozygous dominant mutations in *DNM2* are associated with both intermediate (CMT-DIB; MIM#606482) [2,3] and axonal (CMT2M) [4] forms. *DNM2* encodes the ubiquitously expressed GTPase dynamin-2 that regulates endocytosis, intracellular trafficking, and cytoskeletal dynamics [5,6,7]. Approximately ten different pathogenic *DNM2*-CMT mutations have been identified to date, predominantly clustered within the pleckstrin homology domain, responsible for binding membrane phosphoinositides. These mutations are believed to impair lipid binding, leading to reduced GTPase activity and defective membrane fission [8,9,10,11].

Patients with *DNM2*-CMT mutations exhibit slowed nerve conduction velocities, structural nerve abnormalities, progressive muscle weakness and atrophy, impaired coordination, and skeletal deformities [12]. Additional signs may include ptosis, ophthalmoparesis, cataracts, or neutropenia [13,14,15]. Despite its clinical and genetic heterogeneity, no disease-modifying therapies are approved for CMT [16,17], highlighting the urgent need for mechanistic studies and targeted interventions.

The K562E heterozygous missense mutation is the most common, and the *Dnm2^K562E/+^* mouse model recapitulates motor deficits and muscle atrophy, along with mild peripheral nerve defects [10,18,19]. While the initial characterization of this model suggested a primary myopathy [18], early nerve involvement, such as reduced g-ratio in sciatic nerves at 8 weeks of age, was also described [10,19]. These findings raise the unresolved question of whether muscle pathology arises secondarily to nerve dysfunction or whether the mutation exerts a direct, cell-autonomous effect in skeletal muscle [20].

Furthermore, while loss-of-function mutations cause CMT, gain-of-function mutations lead to centronuclear myopathy (CNM) [3,21], a primary muscle disorder characterized by muscle weakness and atrophy, ptosis, ophthalmoplegia, and centralized organelles [3,8,21]. The gain-of-function effect of CNM mutations was confirmed by studies showing that DNM2 overexpression in wild-type (WT) mice induces a CNM-like phenotype [22,23]. Genetic studies in mice combining CMT and CNM mutations have shown that rebalancing DNM2 activity toward normal levels can prevent the development of both diseases [10]. Moreover, reducing DNM2 protein levels in CNM mouse models with different methodologies ameliorated disease phenotypes [24,25,26,27]. Conversely, whether increasing DNM2 expression can benefit CMT without inducing CNM-like pathology has never been tested.

To elucidate the muscle contribution to the pathogenesis underlying *DNM2*-CMT and to evaluate the therapeutic potential of DNM2 overexpression, we tested two approaches in the *Dnm2^K562E/+^* CMT mouse model: (1) muscle-specific DNM2 overexpression from embryogenesis and (2) postnatal delivery of human DNM2 via adeno-associated virus (AAV) vectors. Muscle-specific DNM2 overexpression initiated during embryogenesis improved several behavioral outcomes and most molecular and histological features in the *Dnm2^K562E/+^* model. In contrast, systemic delivery of human DNM2 at birth increased DNM2 levels in muscle but not in nerve tissue and failed to improve, and in some cases worsened, muscle phenotypes. These findings identify skeletal muscle as a direct contributor to *DNM2*-CMT pathogenesis and demonstrate that both the level and timing of DNM2 expression critically determine therapeutic outcomes. This work provides crucial guidance for the design of safe and effective gene-based interventions and underscores the potential risks of overexpression-based therapeutic approaches.

## 2. Results

### 2.1. Muscle-Specific DNM2 Overexpression from Embryogenesis Improves Dnm2-CMT Locomotor Coordination

The heterozygous K562E mutation, which causes CMT in humans, behaves as a loss-of-function allele with an additional dominant-negative effect in mice, as heterozygous *Dnm2* knockout animals do not recapitulate the neuromuscular phenotypes seen in *Dnm2^K562E/+^* mice [18,28]. To investigate the therapeutic potential of increasing WT DNM2 levels, we first intended to cross *Dnm2^K562E/+^* mice with a transgenic (Tg) line ubiquitously (Ub) overexpressing murine DNM2 (TgDNM2^Ub^) in which DNM2 expression is triggered by expression of the Cre recombinase under the control of the ACTB promoter. However, TgDNM2^Ub^ mice exhibited perinatal lethality. Although embryos were detected at embryonic day 18.5 (E18.5), no viable pups with this genotype were identified at postnatal day 10 (P10) (Supplementary Figure S1A,B).

**Figure 1:**
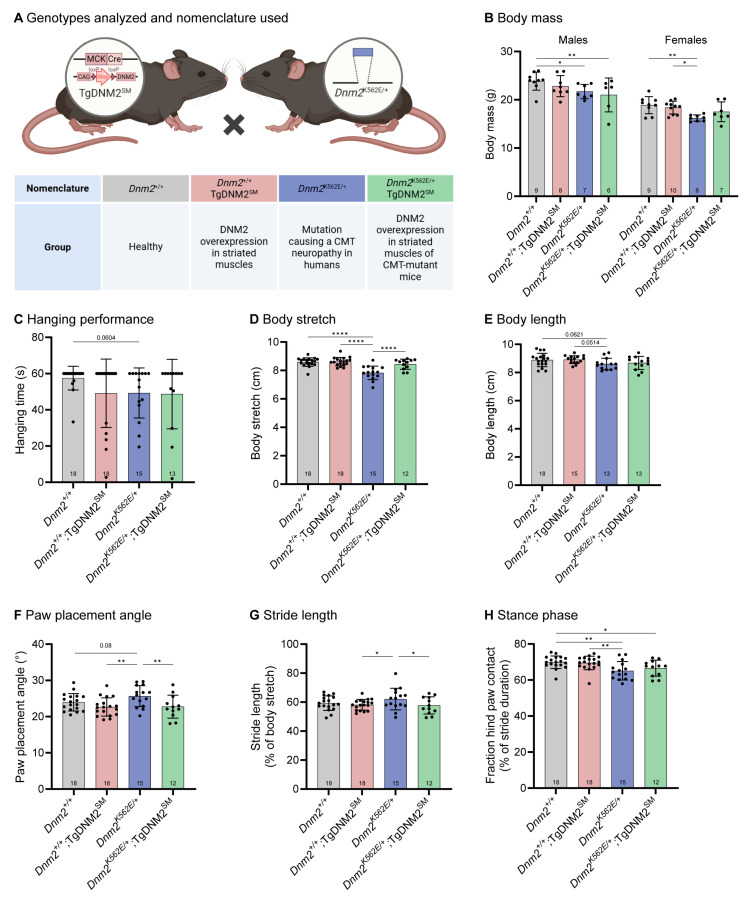
Muscle-specific DNM2 overexpression from embryogenesis improves *Dnm2*-CMT locomotor coordination. (**A**) Genotypes analyzed and nomenclature used. Created with BioRender. (**B**) Body mass of males (6 ≤ *n* ≤ 9) and females at 8 w (7 ≤ *n* ≤ 10). (**C**) Hanging test performance at 8 w. Maximum hanging time = 60s (13 ≤ *n* ≤ 18). (**D**) Body stretch (nose to tail base length) measured during treadmill walking at 8 w (12 ≤ *n* ≤ 18). (**E**) Body length (nose to tail base) measured after euthanasia at 8 w (13 ≤ *n* ≤ 18). (**F**) Angle of feet between paw and body line during treadmill walking (12 ≤ *n* ≤ 18). (**G**) Stride (= length of a step) during treadmill walking normalized to body stretch (12 ≤ *n* ≤ 18). (**H**) Stance phase (=fraction of each stride during which the hind paw remains in contact with the treadmill) (12 ≤ *n* ≤ 18). In (**A**), X indicates crossing. Each dot represents a mouse. Values are represented as mean ± SD, * *p* < 0.05, ** *p* < 0.01, **** *p* < 0.0001. (**B**,**D**–**G**) ANOVA test. (**C**,**H**) Kruskal–Wallis test.

Therefore, as a main affected tissue in the *Dnm2^K562E/+^* mouse is muscle, and to further elucidate the impact of the K562E CMT mutation within this tissue, we restricted the overexpression of DNM2 specifically in striated muscles (SM) upon expression of the Cre recombinase under the control of the muscle-specific MCK promoter by crossing *Dnm2^K562E/+^* mice with TgDNM2^SM^ transgenic mice. The resulting *Dnm2^K562E/+^*;TgDNM2^SM^ mice were viable, and we assessed their growth and locomotor function at 8 weeks (Figure 1A). RT-qPCR showed a 3.2- and 2.1-fold increase in *Dnm2* RNA in tibialis anterior (TA) muscle from *Dnm2^+/+^*;TgDNM2^SM^ and *Dnm2^K562E/+^*;TgDNM2^SM^ mice, respectively, compared to the endogenous DNM2 levels in non-overexpressing controls, while western blotting showed a 3.8- and 4.8-fold increase in protein expression (Supplementary Figure S2A,B). Some inter-animal variability was observed, likely reflecting mosaic MCK-Cre activity and variable recombination efficiency.

*Dnm2^K562E/+^* mice showed reduced body mass compared to controls, which was not rescued in males and showed a tendency toward improvement in females following DNM2 overexpression (not different from disease nor control groups) (Figure 1B). As no sex differences were reported in the *Dnm2^K562E/+^* mouse model [10], data from males and females were pooled for all analyses, except for body mass. Although not statistically significant, the *Dnm2^K562E/+^* mice exhibited a decrease in hanging time (*p* = 0.0604, Figure 1C), which was not modified upon DNM2 overexpression. A previous study reported no intrinsic muscle weakness in *Dnm2^K562E/+^* mice during in situ muscle force measurement [10], further supporting the absence of a primary contractile deficit. When walking on the treadmill, *Dnm2^K562E/+^* mice appeared smaller, as indicated by a significant reduction in body stretch (Figure 1D), likely attributable to smaller overall body size (Figure 1E, not significant). Of note, body stretch was fully rescued following DNM2 overexpression, despite no increase in body length, suggesting a functional improvement in muscle tone rather than overall growth. *Dnm2^K562E/+^* mice displayed increased paw placement angle and stride length, reflecting spatial gait impairments and coordination deficits, both of which were rescued by muscle-specific DNM2 overexpression (Figure 1F,G). By contrast, the reduced stance phase—the fraction of each stride during which the hind paw contacts the treadmill—suggests instability, impaired postural control, or compensatory strategies and was not improved by DNM2 overexpression (Figure 1H).

Overall, muscle-specific DNM2 overexpression rescues body stretch and spatial gait defects but does not correct temporal gait parameters, indicating partial recovery of locomotor coordination in *Dnm2^K562E/+^* mice.

### 2.2. Muscle-Specific DNM2 Overexpression Does Not Improve Dnm2-CMT Muscle Atrophy

To better understand the motor improvement observed, we analyzed muscle mass and histology. Both TA and Soleus muscle masses were decreased in *Dnm2^K562E/+^* mice compared to controls (Supplementary Figure S2C,D). DNM2 overexpression did not improve TA mass and showed only a non-significant trend toward improvement in Soleus mass. Histological analysis of TA muscle revealed myofiber hypotrophy, with a 1.2-fold higher proportion of small fibers compared to controls, which was not rescued by DNM2 overexpression (Supplementary Figure S2E–G). No significant increase in centralized nuclei was observed following DNM2 overexpression (Supplementary Figure S2H).

Overall, muscle-specific DNM2 overexpression improved or fully rescued several gait defects, including the body stretch, paw placement and stride length of *Dnm2^K562E/+^* mice, but did not rescue muscle atrophy.

### 2.3. Muscle-Specific DNM2 Overexpression Partially Improves Dnm2-CMT Muscle Organization

**Figure 2:**
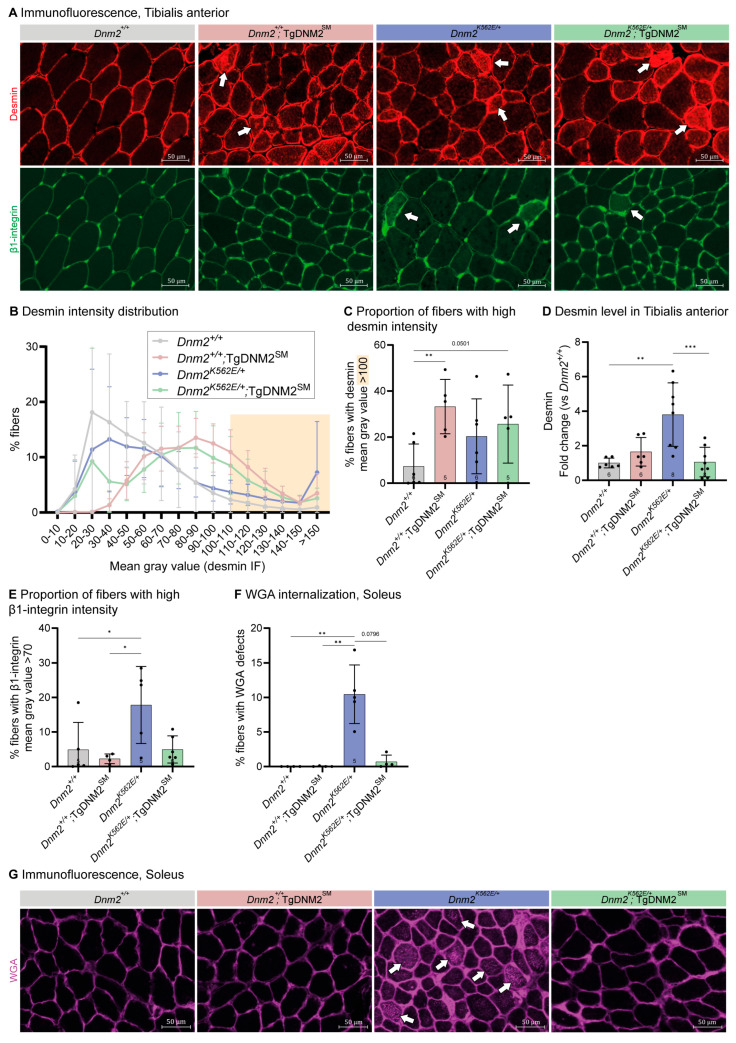
Muscle-specific DNM2 overexpression partially improves *Dnm2*-CMT muscle organization. (**A**) Immunolabeling of desmin (top panel) and β1-integrin (second panel) in transversal Tibialis anterior (TA) sections. Arrows indicate examples of fibers presenting an abnormal central accumulation of the staining. Scale bar = 50 µm. (**B**) TA fibers distribution based on their desmin fluorescence intensity (the higher the gray value, the brighter the fiber) (5 ≤ *n* ≤ 6). (**C**) Proportion of fibers with high desmin intensity (mean gray value > 100) (5 ≤ *n* ≤ 6). (**D**) Quantification of Desmin protein level in TA at 8 w, normalized to Ponceau S staining, depicted in Supplementary Figure S3A (6 ≤ *n* ≤ 8). (**E**) Proportion of fibers with high integrin intensity (mean gray value > 70) (4 ≤ *n* ≤ 6). (**F**) Proportion of fibers presenting an abnormal localization of WGA in Soleus sections at 8 w (4 ≤ *n* ≤ 5). (**G**) Labeling of transversal Soleus sections with fluorescent WGA. Arrows indicate examples of fibers presenting an abnormal central accumulation of the staining. Each dot represents a mouse. Values are represented as mean ± SD, * *p* < 0.05, ** *p* < 0.01, *** *p* < 0.001. (**D**) ANOVA test. (**C**,**E**,**F**) Kruskal–Wallis test.

Since DNM2 overexpression improved locomotor coordination but not muscle atrophy in *Dnm2^K562E/+^* mice, we next examined the molecular and cellular bases of this apparent discrepancy. Desmin, a key cytoskeletal protein that was shown to interact [29] and colocalize with DNM2 [23] and to be disrupted by *DNM2* mutations or depletion [10,26,29], was examined to assess muscle organization. Desmin immunofluorescence in transversal TA sections revealed altered intensity distributions (Figure 2A, upper panel, white arrows). Control muscles had a high proportion of low-intensity fibers. *Dnm2^K562E/+^* muscles had ~20% of fibers exhibiting high intensity (mean gray value > 100), compared to ~7% in WT controls, though this difference was not statistically significant (Figure 2B,C), suggesting impaired protein turnover. This higher proportion of fibers with high desmin intensity was not rescued in *Dnm2^K562E/+^*;TgDNM2^SM^, while *Dnm2^+/+^*;TgDNM2^SM^ muscles showed a shift toward higher intensities. The tendency of desmin mislocalization in *Dnm2^K562E/+^* muscle correlated with increased desmin protein levels, which were completely normalized following DNM2 overexpression (Figure 2D, Supplementary Figure S3A).

Similarly, β1-integrin, an adhesion molecule that traffics in a dynamin-dependent pathway and links the cytoskeleton to the extracellular matrix (ECM) [30,31], showed abnormal intracellular staining in some *Dnm2^K562E/+^* fibers (Figure 2A, lower panel, white arrows). Analysis of fibers with high β1-integrin staining intensity (mean gray value > 70) revealed an increase in *Dnm2^K562E/+^* mice (18%) compared to controls (5%) (Figure 2E). Overexpression of DNM2 in *Dnm2^K562E/+^*; TgDNM2^SM^ muscles restored the proportion of high-intensity fibers to control levels (5%). In addition, immunofluorescence of wheat-germ-agglutinin (WGA), which labels surface glycoproteins and glycolipids, revealed abnormal internal staining inside ~10% of fibers in *Dnm2^K562E/+^* sections in Soleus muscle (Figure 2F,G, white arrows). WGA fluorescence has been previously used to assess membrane internalization in muscle cells [32]. Together with integrin findings, these results support impaired membrane trafficking in *Dnm2^K562E/+^* muscle. WGA internalization was fully rescued in *Dnm2^K562E/+^*;TgDNM2^SM^ overexpressing DNM2.

Prior transcriptomic and histological analyses of Soleus muscle revealed increased ECM deposition in *Dnm2^K562E/+^* mice [18,19], a finding also observed in *DNM2*-CMT patient [14]. We thus assessed collagen VI expression and found increased collagen thickness between TA muscle fibers in *Dnm2^K562E/+^* mice (Supplementary Figure S3B,D), confirmed by Masson’s Trichrome staining showing fibrosis in the Soleus muscle (Supplementary Figure S3C,E, white arrows). While DNM2 overexpression did not improve fibrosis in Soleus, it fully normalized collagen thickness in TA muscle.

Overall, muscle-specific DNM2 overexpression corrected key defects in cytoskeletal organization, membrane dynamics, and extracellular matrix structure in *Dnm2^K562E/+^* mice that potentially explain the rescue of body stretch, paw placement, and stride length despite a conserved muscle atrophy.

### 2.4. Muscle-Specific DNM2 Overexpression Markedly Improves Dnm2-CMT Mitochondrial Dysfunction

**Figure 3:**
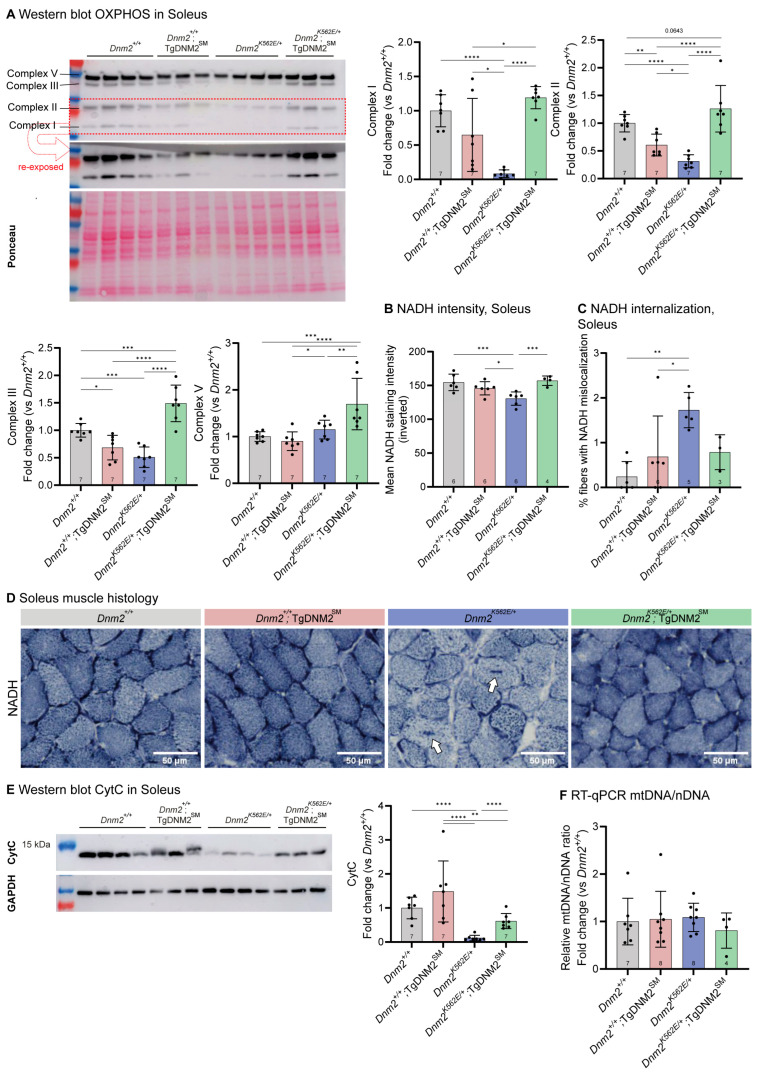
Muscle-specific DNM2 overexpression markedly improves *Dnm2*-CMT mitochondrial dysfunction. (**A**) Representative western blot and quantification of complexes I, II, III, and V of the electron transport chain, assessed using the OXPHOS antibody cocktail in Soleus (*n* = 7). (**B**) Mean NADH staining intensity in Soleus muscle fibers, shown as inverted grayscale values (255-raw intensity), so that higher values correspond to darker staining (4 ≤ *n* ≤ 6). (**C**) Proportion of fibers displaying internalized NADH staining (3 ≤ *n* ≤ 6). (**D**) Soleus transversal sections stained for nicotinamide adenine dinucleotide (reduced form) (NADH); arrows indicate fibers with abnormal central accumulation of staining. Scale bar = 50 µm. (**E**) Quantification of cytC protein level in Soleus, normalized to Ponceau S staining, depicted in Supplementary Material (*n* = 7). (**F**) Mitochondrial DNA (mtDNA) to nuclear DNA (nDNA) ratio in Soleus muscle, quantified by RT-qPCR using *Nd1* and *Rsp11,* respectively (4 ≤ *n* ≤ 8). Each dot represents a mouse. Values are represented as mean ± SD, * *p* < 0.05, ** *p* < 0.01, *** *p* < 0.001, **** *p* < 0.0001. (**A**,**B**,**E**) ANOVA test. (**C**,**F**) Kruskal–Wallis test.

Previous transcriptomic and molecular analyses of Soleus muscle revealed mitochondrial dysregulation in *Dnm2^K562E/+^* mice [18,19], which may underlie part of the gait defects of *DNM2*-CMT. We thus assessed the expression of electron transport chain complexes by western blot and found that Complexes I, II, and III were downregulated in *Dnm2^K562E/+^* mice (Figure 3A). DNM2 overexpression fully restored the levels of the affected complexes. Complex I (NADH dehydrogenase) reduction in *Dnm2^K562E/+^* mice correlated with a decreased NADH staining intensity in muscle fibers and the presence of abnormal internal staining in some fibers (Figure 3B–D). DNM2 overexpression normalized NADH staining intensity and improved staining localization. Complex II (succinate dehydrogenase) reduction did not associate with SDH staining abnormalities, as previously reported in TA [19]. In contrast, Complex III (ubiquinol-cytochrome c oxidoreductase) reduction was associated with a marked decrease in cytochrome c protein levels (Figure 3E), which was fully normalized by DNM2 overexpression. Importantly, these changes were not due to reduced mitochondrial content (Figure 3F). These findings indicate a strong correction of mitochondrial abnormalities at the level of protein abundance and enzymatic staining. Improved mitochondrial homeostasis likely enhances muscle energy metabolism, which may contribute to the gait improvements observed despite persistent atrophy.

We observed a shift toward faster fiber types in the Soleus in *Dnm2^K562E/+^* mice, with a reduced proportion of type I fibers and an increased proportion of type IIa fibers (Supplementary Figure S3F). DNM2 upregulation fully rescued the proportion of type I fibers and partially restored type IIa levels. These changes may explain the overall mitochondrial abnormalities observed in *Dnm2^K562E/+^* mice and their rescue following DNM2 upregulation.

In conclusion, muscle-specific DNM2 overexpression (4.8-fold increase vs. WT controls, 4.1-fold vs. *Dnm2^K562E/+^*) in *Dnm2^K562E/+^*;TgDNM2^SM^ mice improved gait parameters but failed to rescue TA muscle atrophy. However, it significantly corrected desmin protein levels and improved membrane trafficking defects (abnormally high integrin intensity and WGA internalization). It also normalized collagen VI thickness in TA and improved mitochondrial homeostasis in the Soleus.

Overall, these data reveal a primary involvement of skeletal muscle in the *DNM2*-CMT pathology and support early DNM2 augmentation as a potential therapeutic strategy.

### 2.5. Postnatal DNM2 Overexpression Does Not Improve Dnm2-CMT Whole-Body Motor Performance

**Figure 4:**
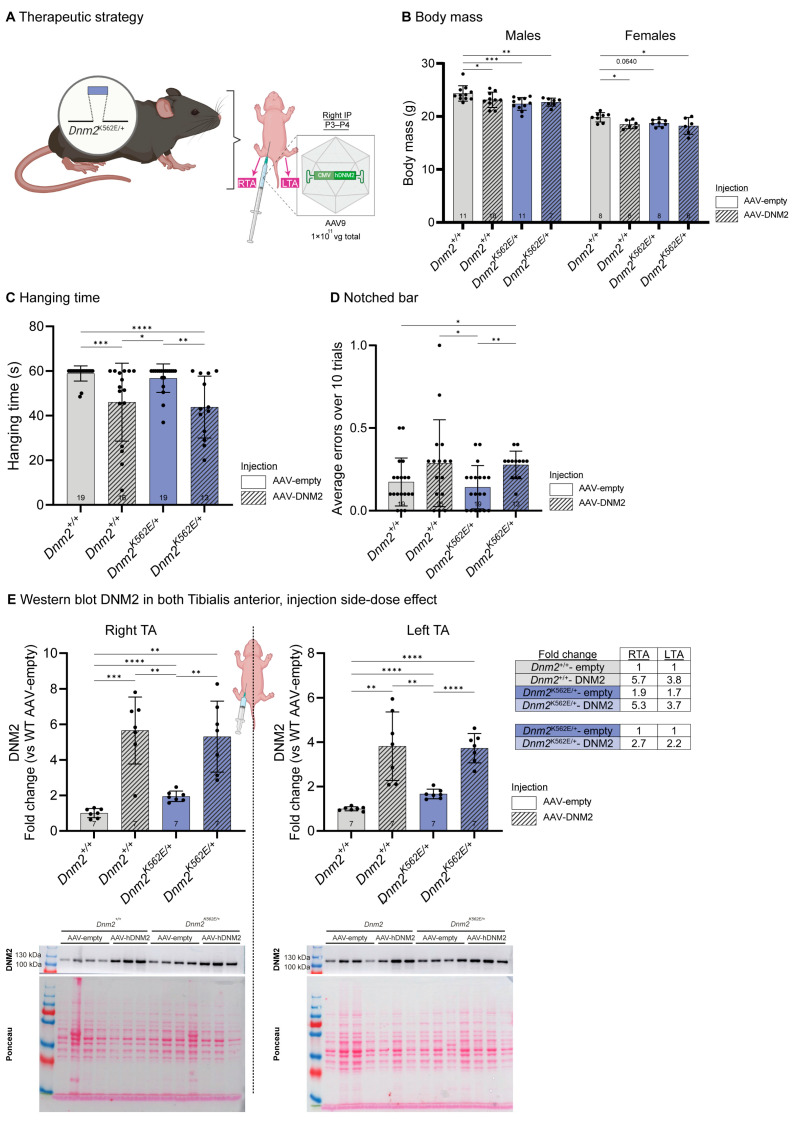
Postnatal DNM2 overexpression does not improve *Dnm2*-CMT whole-body motor performance. (**A**) Experimental set-up. Created with BioRender. (**B**) Body mass of males (7 ≤ *n* ≤ 11) and females at 8 w (6 ≤ *n* ≤ 8). (**C**) Hanging test performance at 8 w. Maximum hanging time = 60 s (13 ≤ *n* ≤ 19). (**D**) Average number of errors (hindlimb slips) when crossing the notched bar over 10 trials (13 ≤ *n* ≤ 19). (**E**) Representative western blot and quantification of DNM2 protein in right TA (IP-injected side) and left TA (contralateral side), normalized to Ponceau S staining (*n* = 7). The summary table reports the fold change in DNM2 levels for each bodyside, relative to untreated *Dnm2^+/+^* and *Dnm2^K562E/+^* groups. Each dot represents a mouse. Values are represented as mean ± SD, * *p* < 0.05, ** *p* < 0.01, *** *p* < 0.001, **** *p* < 0.0001. (**B**,**E**) ANOVA test. (**C**,**D**) Kruskal–Wallis test.

As increasing DNM2 levels in striated muscles from embryogenesis showed beneficial effects in the *Dnm2^K562E/+^* model, we next explored a translational gene therapy approach aimed at preventing disease onset. To reach systemic transduction and assess therapeutic efficacy once embryogenesis was finalized, we administered AAV9 vectors carrying human DNM2 under the ubiquitous CAG promoter via intraperitoneal injection at birth at a dose of 1.0 × 10^11^ genome copies (gc) per pup and evaluated outcomes at 8 weeks of age (Figure 4A). This route was chosen based on previous reports showing efficient muscle transduction of AAV vectors delivered intraperitoneally [33,34].

The 3.6-fold increase in *DNM2* RNA level in *Dnm2^K562E/+^* mice injected with AAV-DNM2 (Supplementary Figure S4A) did not improve the body mass decrease (Figure 4B). Postnatal DNM2 overexpression worsened the hanging abilities of *Dnm2^+/+^* and *Dnm2^K562E/+^* mice compared to the untreated groups (Figure 4C). DNM2 overexpression also induced coordination defects in *Dnm2^K562E/+^* mice, with an increased number of errors when crossing the notched bar (Figure 4D).

Noteworthy, the intraperitoneal injection was consistently made on the right side in all mice. Western blot analysis showed an average 5.5-fold increase in DNM2 protein levels in the right TA (RTA, IP injection side) of *Dnm2^+/+^* and *Dnm2^K562E/+^* mice, compared to untreated *Dnm2^+/+^*. A lower level of overexpression was observed in the contralateral left TA, averaging a 3.8-fold increase (Figure 4E, Supplementary Figure S4B). This side-dependent difference in DNM2 expression, resulting from the unilateral intraperitoneal injection, enabled evaluation of two distinct doses within the same animal in subsequent analyses.

### 2.6. Postnatal DNM2 Overexpression Does Not Rescue Dnm2-CMT Myofiber Hypotrophy and Promotes CNM Histopathology

Postnatal DNM2 delivery worsened motor performance in *Dnm2^K562E/+^* mice. As intraperitoneal injection at birth produced asymmetric expression, we explored whether higher local DNM2 expression (injected right side ~5.5-fold) could induce myopathic features, consistent with CNM gain-of-function phenotypes, while lower expression (contralateral left side ~3.8-fold) might be beneficial. We therefore analyzed muscle structure and organization in both hindlimbs.

**Figure 5:**
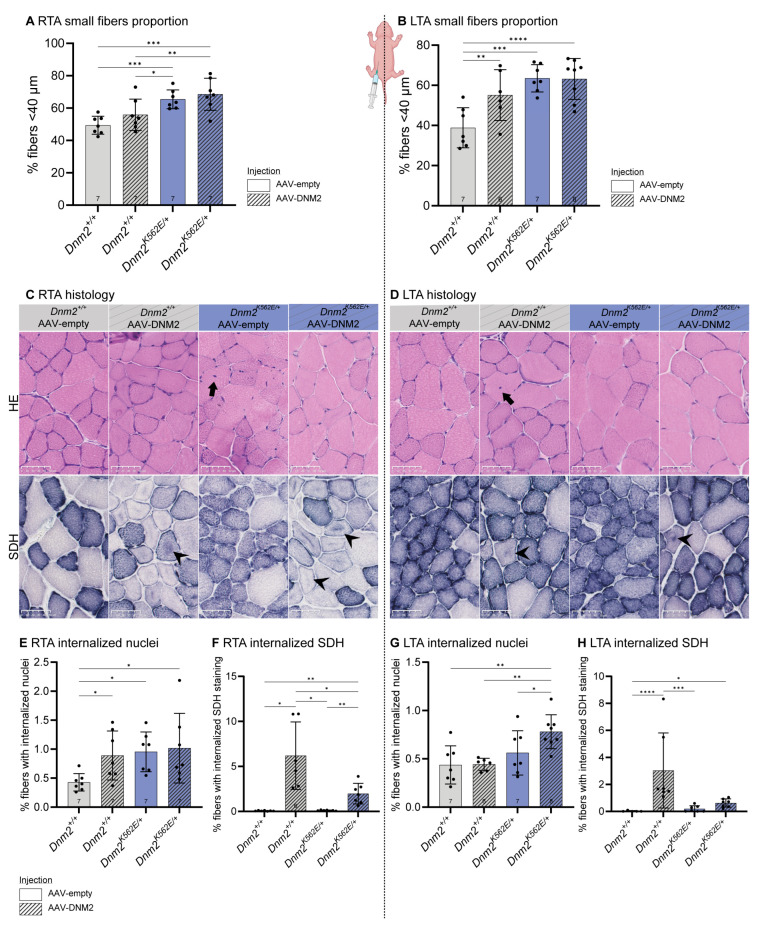
Postnatal DNM2 overexpression does not rescue *Dnm2*-CMT myofiber hypotrophy and promotes CNM histopathology. (**A**,**B**) Proportion of small fibers (MinFeret < 40 µm) in (**A**) right TA (RTA, IP-injected side) (*n* = 7) and (**B**) left TA (LTA, contralateral side) (6 ≤ *n* ≤ 8). (**C**,**D**) Transversal sections of TA stained with hematoxylin and eosin (HE, top panel) and succinate dehydrogenase (SDH, second panel) in (**C**) right TA (IP-injected side) and (**D**) left TA (contralateral side). Scale bar = 50 µm. Black arrows indicate internalized nuclei; black arrowheads indicate internalized oxidative staining. (**E**,**F**) Proportion of fibers with (**E**) internalized nuclei and (**F**) internalized oxidative staining in right TA (IP-injected side) (6 ≤ *n* ≤ 7). (**G**,**H**) Proportion of fibers with (**G**) internalized nuclei and (**H**) internalized oxidative staining in left TA (contralateral side) (6 ≤ *n* ≤ 8). Each dot represents a mouse. Values are represented as mean ± SD, * *p* < 0.05, ** *p* < 0.01, *** *p* < 0.001, **** *p* < 0.0001. (**A**–**G**) ANOVA test. (**H**) Kruskal–Wallis test.

TA muscle mass, which was reduced in *Dnm2^K562E/+^* mice, was restored to *Dnm2^+/+^* levels on both sides following DNM2 delivery (Supplementary Figure S4C,D). However, this recovery did not translate into improved muscle fiber morphology. Myofiber hypotrophy persisted, with an elevated proportion of small-diameter fibers in *Dnm2^K562E/+^* mice on both the right TA (IP-injected side, ~5.5-fold) and left TA (contralateral side, ~3.8-fold) (Figure 5A,B), suggesting that the muscle mass increase results from ECM expansion or fat infiltration rather than myofiber hypertrophy.

Histological analysis revealed abnormalities linked to DNM2 overexpression. In particular, hematoxylin-eosin (HE) staining revealed that nuclear mispositioning (Figure 5C,D, first panel, black arrows) was exacerbated in *Dnm2^+/+^* mouse RTA (Figure 5E) and in *Dnm2^K562E/+^* mouse LTA (Figure 5G). Similarly, succinate dehydrogenase (SDH) staining revealed disorganized mitochondrial positioning (Figure 5C,D, second panel, black arrowheads) induced by DNM2 overexpression in both genotypes, particularly in the right TA (Figure 5F,H). Myofiber hypotrophy combined with increased nuclear and mitochondrial mispositioning recapitulated the main histopathological hallmarks of CNM following postnatal DNM2 overexpression.

### 2.7. Postnatal DNM2 Overexpression Fails to Restore Desmin and Integrin Localization in Dnm2-CMT Muscle

**Figure 6:**
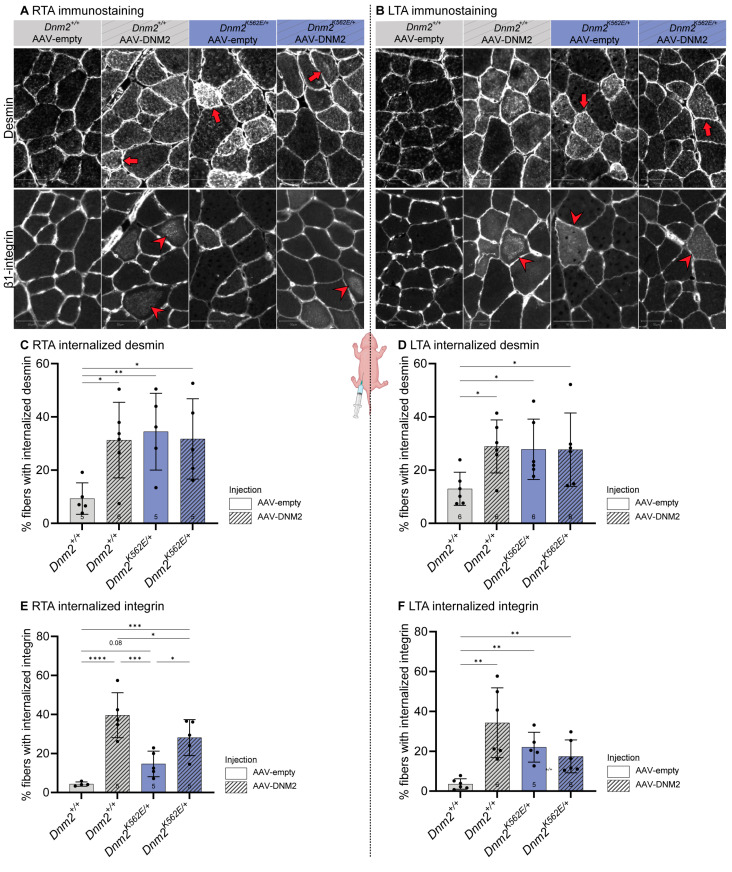
Postnatal DNM2 overexpression fails to restore desmin and integrin localization in *Dnm2*-CMT muscle. (**A**,**B**) Transversal sections of TA immunolabelled for desmin (top panel) or β1-integrin (second panel) in (**A**) right TA (IP-injected side) and (**B**) left TA (contralateral side). Scale bar = 50 µm. Red arrows and arrowheads mark fibers with abnormal central accumulation of desmin and β1-integrin staining, respectively. (**C**,**D**) Proportion of fibers presenting an abnormal localization of desmin in (**C**) right TA (IP-injected side) (5 ≤ *n* ≤ 6) and (**D**) left TA (contralateral side) at 8 w (*n* = 6). (**E**,**F**) Proportion of fibers presenting an abnormal localization of β1-integrin in (**E**) right TA (IP-injected side) (4 ≤ *n* ≤ 5) and (**F**) left TA (contralateral side) (5 ≤ *n* ≤ 6). Each dot represents a mouse. Values are represented as mean ± SD, * *p* < 0.05, ** *p* < 0.01, *** *p* < 0.001, **** *p* < 0.0001. (**C**–**F**) ANOVA test.

We next assessed desmin and β1-integrin localization by immunofluorescence, given their previously observed mislocalization in *Dnm2^K562E/+^* muscle and rescue following embryonic DNM2 overexpression. Desmin accumulated abnormally in ~31% of *Dnm2^K562E/+^* TA fibers, compared to ~11% in controls, and this defect was not rescued by either side of AAV-DNM2 injection (Figure 6A,B, first panel, red arrows; Figure 6C,D). Similarly, integrin was mislocalized inside myofibers in ~18% of *Dnm2^K562E/+^* fibers versus ~4% in *Dnm2^+/+^*. AAV-DNM2 worsened this defect, with the right TA showing increased mislocalization in both genotypes, as did the left TA in *Dnm2^+/+^*, without benefit in *Dnm2^K562E/+^* (Figure 6A,B, second panel, red arrowheads; Figure 6E,F). As postnatal intraperitoneal AAV-DNM2 injection did not increase DNM2 levels in the sciatic nerve at 8 weeks on either body side, nerve analysis was not pursued (Supplementary Figure S4E,F).

## 3. Discussion

This study investigated the contribution of skeletal muscle to the peripheral neuropathy caused by *DNM2* loss-of-function mutations and assessed the therapeutic potential of increasing DNM2 expression. We uncovered a primary involvement of skeletal muscle in *DNM2*-CMT pathology. Muscle-specific DNM2 overexpression from embryogenesis partially improved muscle organization and gait parameters, whereas postnatal delivery failed to rescue the pathology and instead induced a CNM-like phenotype (summary in Supplementary Figure S5). Collectively, these findings highlight the narrow therapeutic window for safe and effective DNM2-based interventions, both in terms of developmental stage and expression level.

### 3.1. A Dual Tissue Pathomechanism in DNM2-Related CMT

Our findings provide new insight into the dual-tissue pathomechanism of *DNM2*-related CMT. We found the *Dnm2^K562E/+^* mouse model exhibits muscle fiber hypotrophy, fibrosis, mislocalization of cytoskeletal and trafficking proteins, and mitochondrial dysfunction. Muscle-specific DNM2 overexpression from embryogenesis did not rescue fiber atrophy, indicating this defect may be secondary to denervation. However, it fully prevented mitochondrial defects and improved desmin and integrin expression defects, membrane trafficking, and collagen thickness, and it correlated with improved locomotor coordination. Notably, these features were ameliorated in the absence of nerve-targeted intervention, indicating that muscle pathology arises independently and is not merely a secondary consequence of denervation. Of note, muscle biopsies from *DNM2*-CMT patients reveal variable myopathic features. For example, Gastrocnemius biopsies (G359D mutation) show fiber atrophy, fibrosis, and type 2 fiber predominance, while Deltoid biopsies (K559del mutation) display mild oxidative changes with largely normal morphology [13,14]. In addition, the ptosis and ophthalmoplegia signs often noted in *DNM2*-CMT patients are not typical signs of CMT but are hallmarks of *DNM2*-CNM. Although chronic denervation complicates interpretation in patients, our findings strongly support a primary pathogenic role of DNM2 loss-of-function within muscle tissue itself.

### 3.2. DNM2 Dosage Sensitivity and Therapeutic Window in Muscle

DNM2 expression must be tightly regulated, as both loss and excess are pathogenic. Constitutive *Dnm2* knockout is embryonically lethal [28,35], and muscle-specific deletion leads to early postnatal death [36], highlighting the critical role of DNM2 in embryonic viability and muscle development. Conversely, DNM2 overexpression in WT animals induces centronuclear myopathy [23], confirming dose-dependent toxicity. Elevated DNM2 levels have also been found in *DNM2*-CNM patients (unpublished data), as well as in *Dnm2*-CNM and in *Dnm2*-CMT mouse models [10], where they correlate with muscle pathology.

In this study, postnatal intraperitoneal injection of AAV-DNM2 failed to reach peripheral nerves, likely due to the route of administration rather than the vector capsid (AAV9) or promoter (CAG), both of which efficiently transduce nerves when delivered intravenously [37]. This approach, however, allowed us to focus on skeletal muscle and compare two levels of DNM2 overexpression in tibialis anterior muscles, with higher expression on the injection side, likely reflecting a minor contribution of local viral diffusion in addition to systemic delivery. Embryonic, muscle-specific DNM2 overexpression (~4.8-fold) in *Dnm2^K562E/+^*;TgDNM2^SM^ improved muscular defects without toxicity. In contrast, comparable levels of DNM2 achieved postnatally via AAV injection (~3.8 to 5.5-fold) induced CNM-like histopathology in both *Dnm2^+/+^* and *Dnm2^K562E/+^* mice. We cannot exclude that the observed CNM-like phenotypes reflect local overexpression artefacts rather than true dose-dependent effects, and intravenous delivery for symmetric systemic expression was not assessed in this study. Collectively, these findings underscore the importance of DNM2 dosage for neuromuscular homeostasis and indicate a narrow developmental window for therapeutic intervention.

Our findings have important implications for therapeutic development in *DNM2*-CMT. The rationale for increasing WT DNM2 expression in a loss-of-function context is conceptually sound, to increase the overall DNM2 activity and dilute the potential dominant negative effect of the DNM2 mutant. However, our results reveal that while embryonic muscle-targeted DNM2 overexpression partially corrected muscle pathology, postnatal delivery failed to rescue disease phenotypes and, in some cases, induced CNM-like pathology. This paradox, in which efforts to compensate for a loss-of-function neuropathy risk inducing a gain-of-function myopathy, highlights that both timing and dosage are critical determinants of therapeutic efficacy and safety. The CNM-like abnormalities observed following postnatal overexpression suggest that DNM2 may have exceeded a toxic threshold in some fibers, but this is unlikely to be the major driver of pathology, as similar expression levels achieved in transgenic mice improved several phenotypes without toxicity. Thus, the lack of efficacy following postnatal injection is more likely attributable to the timing of DNM2 overexpression; postnatal expression may miss a critical developmental window or interfere with muscle postnatal maturation. Additional factors, such as the need for concurrent nerve targeting or species-specific differences in the human DNM2 isoform used here, may also contribute. It is further possible that increasing DNM2 after birth in a CMT context is simply insufficient to reverse an already established pathology. In contrast, in CNM models, DNM2 overactivity remains reversible postnatally, as antisense oligonucleotide-mediated DNM2 reduction after disease onset can rescue CNM muscle phenotypes [25].

### 3.3. Translational Considerations

While muscle targeting proved effective in this model, CMT patients primarily exhibit neuropathic features. Our findings therefore raise the question of whether combined targeting of both muscle and nerve would be necessary to achieve meaningful clinical outcomes, an aspect difficult to evaluate here due to the mild nerve phenotype of the model. Such nerve-targeting evaluation would require optimizing the route of AAV delivery and capsid choice for efficient nerve transduction [38,39]. Moreover, as postnatal preventive delivery failed in mice, and as patients are typically diagnosed after symptom onset, future work should assess alternative dosing strategies or timing of intervention. Given the dose-sensitive nature of DNM2, modulating its activity rather than its expression level, or employing allele-specific knockdown approaches [40], may offer safer and more flexible therapeutic alternatives.

## 4. Materials and Methods

### 4.1. Mouse Models

The ACTB-Cre^+^ line was on a 100% C57BL/6N background. TgDNM2^SM^ mice were generated by crossing MCK-Cre^+^ mice (expressing Cre recombinase in striated muscle from embryonic day 12.5; 100% C57BL/6N, provided by Hélène Puccio) with CAG-LSL-DNM2 mice. The latter carry a loxP-flanked STOP cassette upstream of the murine *Dnm2* gene (transcript variant 3, NM_007871.2), driven by the ubiquitous CAG promoter and inserted into the Rosa26 locus (generated at ICS; project IR8041/Kos8041; 100% C57BL/6N). Cre-mediated recombination results in muscle-specific expression of DNM2, from embryonic day 13 (E13) [41]. These mice were then crossed with *Dnm2^K562E/+^* knock-in mice (100% C57BL/6J; previously described [18]) to generate experimental cohorts. All mice used in the study, including controls, carried the MCK-Cre, were of mixed genetic background (50% C57BL/6N and 50% C57BL/6J), included both sexes, and were analyzed at 8 weeks of age.

For genotyping, primers “6115 Er KE” and “6116 Ef KE” were used to detect K562E mutation, primers “Cre 160” and “Cre 161” to detect the Cre, and “Sf 10692” “Wr 4035” for the CAG-LSL-*Dnm2* transgene (Supplementary Table S1 Reagents).

Mice were housed in ventilated cages, with unrestricted access to food and water. Environmental parameters were maintained at 19–22 °C temperature and 40–60% relative humidity, under a 12 h light/dark cycle. Breeding animals were fed with SAFE^®^ D03 diet (SAFE, Augy, France), transitioning to SAFE^®^ D04 after weaning.

### 4.2. AAV Vector Design and Production

Recombinant AAV9 vectors were produced by the Molecular Biology and Virus Facility at IGBMC using a standard triple transfection protocol in HEK293T/17 cells. Expression plasmid encoding human *DNM2* (transcript variant 3, NCBI RefSeq NM_004945.4) cDNA (excluding exons 12b and 13ter) was cloned under the control of the ubiquitous CAG promoter. This construct, pAAV-CAG-hDNM2 and the empty control pAAV-mU6-MCS empty, were co-transfected with the pHelper plasmid (Agilent, Santa Clara, CA, USA) and the pAAV2/9 capsid plasmid (P0008, Penn Vector Core, Philadelphia, PA, USA).

Viral particles were harvested 48 h post-transfection from Benzonase-treated (100 U/mL, Merck, Darmstadt, Germany) cell lysates. Purification was performed via iodixanol gradient ultracentrifugation (OptiPrep™, Serumwerk Bernburg AG, Bernburg, Germany), followed by dialysis and concentration in Dulbecco’s PBS supplemented with 0.5 mM MgCl_2_, using Amicon Ultra-15 centrifugal filters (100 kDa cutoff, Merck Millipore).

Final titers were determined by quantitative PCR (qPCR) using LightCycler 480 SYBR Green I Master mix (Roche Diagnostics, Basel, Switzerland), with primers specific to hDNM2 or the CMVe enhancer (Supplementary Table S1 Reagents). Purified vectors were aliquoted and stored at −80 °C until use.

### 4.3. In Vivo AAV Injections

Intraperitoneal injections were performed in neonatal mice (P3–P5). A total volume of 50 µL of AAV9 vector (2.0 × 10^12^ gc/mL) was injected into the lower right abdominal quadrant using a 31G syringe, corresponding to a dose of 1.0 × 10^11^ gc per pup. For untreated controls, equivalent volumes and concentrations of AAV-empty vectors were administered using the same injection protocol.

### 4.4. Behavioral Tests

Behavioral assessments were conducted at 8 weeks of age, by a single experimenter blinded to genotype and treatment. The hanging test measured latency to fall from an inverted grid (max 60 s); three trials were performed per animal, and the two best were averaged. Gait analysis was performed on a motorized treadmill equipped with a camera, at a constant speed of 14 cm/s. Body stretch (nose to tail base when hind paw was in contact with the surface), stride length (step distance normalized to body stretch), and paw placement angle (angle between body axis and paw center when in contact with the surface) were quantified. Three measures were averaged for body stretch and stride, and six for paw angle (3/side). The stance phase was calculated as the duration of hind paw contact with the treadmill divided by the total step cycle duration, expressed as a percentage. Three measures were averaged per mouse. Sensorimotor coordination was assessed using the notched bar test, where mice crossed a horizontal bar with alternating notches. The number of hindlimb slips into notches were recorded over 10 trials per mouse and averaged. Body length was measured post-mortem (nose to tail base).

### 4.5. Tissue Collection

Mice were euthanized by carbon dioxide inhalation. TA and Soleus muscles were dissected, weighed, and snap-frozen in liquid nitrogen-cooled isopentane and stored at −80 °C for histological, RNA, or protein analyses. Sciatic nerves were snap-frozen in liquid nitrogen and stored at −80 °C.

### 4.6. Muscle Histology

Transverse TA and soleus sections with a thickness of 8 µm were cut using a cryostat and stained with HE, SDH, NADH, and Masson’s trichrome by the histology platform. Slides were scanned using the Carl Zeiss Axioscan 7 (Zeiss, Oberkochen, Germany), and 20x images were used for analysis.

Fiber segmentation was performed on HE stained sections using CellPose software (version 0.6.1) [42]. MinFeret diameter were quantified in Fiji (version 2.14.0/1.54f, ImageJ) [43]. Central nuclei, SDH and NADH internalization were manually assessed using Fiji or QuPath (version 0.5.1) [44]. NADH intensity was quantified as the mean gray value per fiber (scale 0–255), and the average per mouse section was plotted. Fibrotic area in Masson’s trichrome-stained sections was quantified using Fiji, adjusting color thresholds and saturation to identify blue-stained fibrosis, and normalized to the total area. One transversal section of the whole muscle was quantified for each animal.

### 4.7. Muscle Immunofluorescence

Transversal 8 µm TA and Soleus muscle sections were fixed in 4% paraformaldehyde for 20 min, permeabilized with PBS containing 0.2% Triton X-100 (Merck #T8787-250ML) for 10 min, and blocked for 1 h in PBS with 0.1% Triton X-100 and 5% BSA (MP Biomedicals, Santa Ana, CA, USA; #02160069-CF). Sections were incubated overnight at 4 °C with primary antibodies against integrin-β1, desmin, or collagen VI. For fiber-type identification, non-permeabilized transversal sections of Soleus muscle were incubated with antibodies against MYH7, MYH2, and MYH4 (Supplementary Table S1 Reagents). Alexa Fluor-conjugated secondary antibodies or WGA, along with DAPI, were applied for 1 h at room temperature (RT). Slides were mounted using ProLong Gold Antifade reagent (ThermoFisher Scientific, Waltham, MA, USA; #P36934). Images were acquired at 20x magnification using either a Zeiss Axio Observer 7 microscope (Zeiss, Oberkochen, Germany) or the Carl Zeiss Axioscan 7. A no-primary antibody control was included for each animal.

For integrin-β1 and desmin staining in the *Dnm2^K562E/+^*;TgDNM2^SM^ cohort, fibers were segmented using CellPose, and mean gray intensity per fiber was quantified in Fiji. Results were represented as either intensity distribution or the percentage of high-intensity fibers. In the *Dnm2^K562E/+^* IP-injected cohort, fiber segmentation was also performed with CellPose, and fibers were classified in QuPath as positive or negative for integrin-β1 or desmin using a fixed intensity threshold applied uniformly across samples. For WGA, fibers were segmented with Cellpose, and those with internalized staining were manually counted on Fiji. For collagen VI staining, the thickness of the signal between adjacent fibers was measured using the line tool in Fiji. Approximately 50 interfiber regions were quantified per muscle section, with six animals per genotype. For fiber typing, WGA was used to delineate all fibers, and fiber types I, IIa, and IIb were identified by MYH7, MYH2, and MYH4, respectively. As no type IIb fibers were detected in Soleus, the unstained fibers were classified as type IIx. Fiber types were quantified manually in Fiji and expressed as a percentage of the total fibers per section.

### 4.8. RNA Extraction and RT-qPCR

Total RNA was extracted from TA or Soleus muscles using TRI Reagent (Molecular Research Center, Cincinnati, OH, USA; #TR118) and homogenized with the Precellys^®^ Evolution Touch system (Bertin Technologies, Montigny-le-Bretonneux, France) using two 15 s pulses at 5500 rpm. Complementary DNA was synthesized from 500 ng to 1 µg of RNA using SuperScript IV Reverse Transcriptase (Invitrogen, Carlsbad, CA, USA; #18090010). Quantitative PCR was carried out using SYBR Green Master Mix I (Roche Diagnostics, Basel, Switzerland; #04887352001) and 0.5 µM of gene-specific primers (listed in Supplementary Table S1 Reagents). Reactions were performed in technical triplicates on a LightCycler 480 system (Roche Diagnostics).

For the *Dnm2^K562E/+^*;TgDNM2^SM^ cohort, *Dnm2* gene expression was assessed using “*Dnm2* ex6” and “*Dnm2* ex8” primers, and normalized to the *Rpl27* housekeeping gene. In the Soleus, mtDNA was assessed by *Nd1* and nDNA by *Rps11.* For the IP-injected *Dnm2^K562E/+^* cohort, *Dnm2* gene expression was assessed using “*Dnm2* ex10 m+h” and “*Dnm2* ex13 m+h” primers, recognizing both mouse and human sequences, and normalized to *Rps11*. Relative expression was calculated using the 2^−ΔΔCt^ method and expressed as fold change compared to the respective control group.

### 4.9. Protein Extraction

Muscle and nerve samples were homogenized in their respective RIPA buffers: for muscle, the buffer contained 150 mM NaCl, 50 mM Tris (pH 8), 0.5% sodium deoxycholate, 1% NP-40, and 0.1% SDS; for nerve, it contained 2% SDS, 25 mM Tris (pH 8.0), 95 mM NaCl, 2 mM EDTA, and 0.5% sodium deoxycholate. Both buffers were supplemented with 1 mM PMSF, 1 mM sodium orthovanadate, 5 mM sodium fluoride, and 1X protease inhibitor cocktail. Tissue samples were processed using a Precellys^®^ Evolution Touch tissue homogenizer (Bertin Technologies) with two 20 s cycles at 6000 rpm and cleared by centrifugation. Protein concentrations were determined using the DC Protein Assay Kit (Bio-Rad Laboratories, Hercules, CA, USA; #5000116).

### 4.10. Western Blotting

Proteins were resolved on 10% polyacrylamide gels prepared in-house according to standard protocols, except cytochrome C that was resolved on 15% gels. For muscle samples, 10 µg of protein in 12 µL were loaded per lane; for nerve samples, 7 µg of protein in 20 µL were used. Electrophoresis was performed at 130 V for approximately 1 h. Proteins were transferred to nitrocellulose membranes using the Trans-Blot Turbo RTA Mini Nitrocellulose Transfer Kit (#170-4270, BioRad) for 5–10 min at 2.5 A. Membranes were stained with Ponceau S, then blocked for 1 h in TBS containing 5% nonfat dry milk and 0.1% Tween-20 (TBST; #P2287-500ML, Merck). Membranes were incubated overnight at 4 °C with primary antibodies (Supplementary Table S1 Reagents) diluted in TBST with 5% milk. DNM2 was detected with the homemade antibody, except for the IP-injected *Dnm2^K562E/+^* cohort (Figure 4, Supplementary Figure S4), where #PA5-19800 was used to detect both human and mouse DNM2. After washing, membranes were incubated for 1 h at room temperature with the appropriate HRP-conjugated secondary antibodies. Signal detection was performed using enhanced chemiluminescence reagents (#32209, ThermoFisher Scientific), and images were acquired on an Amersham Imager 600 (GE Healthcare Life Sciences, Marlborough, MA, USA).

Band intensities were quantified using Fiji. Data were normalized to Ponceau staining and the mean value of control group, except CytC that was normalized to GAPDH instead of the Ponceau for practical reasons.

All original, uncropped western blot images used for quantification, including those not shown in the figures and their corresponding loading controls, are available in the Supplementary Material.

### 4.11. Statistical Analysis

All statistical analyses and graph generation were performed using GraphPad Prism (version 10.0.2). As no sex differences were reported in the *Dnm2^K562E/+^* mouse model [10], data from males and females were pooled for all analyses, except for body mass. Sex was balanced across experimental groups as much as possible (see Supplementary Table S2: Statistics). Normality was assessed using the Shapiro–Wilk test, except for large sample sizes where the D’Agostino & Pearson test was applied. For normally distributed data with equal variances, one-way ANOVA was performed, followed by post hoc uncorrected Fisher’s LSD tests for pairwise comparisons. When variances were unequal, a Brown-Forsythe and Welch ANOVA was performed, followed by post hoc Welch’s *t*-tests. When data were log-normally distributed, a log transformation was applied before ANOVA analysis. For non-normally distributed data, the Kruskal–Wallis test was used, followed by post hoc uncorrected Dunn’s tests. All pairwise comparisons were performed, but only statistically significant differences are shown on the graphs. A *p*-value < 0.05 was considered significant. Graphs display individual data points with mean ± SD. A detailed summary of the statistical tests and group sizes is provided in Supplementary Table S2.

## 5. Conclusions

This study demonstrates that increasing DNM2 expression specifically in skeletal muscle from embryogenesis can partially rescue key pathological and functional features of *DNM2*-CMT disease, thereby establishing muscle as a primary and therapeutically relevant target tissue in this model. Moreover, we report a first gene therapy approach aiming to overexpress DNM2 postnatally via systemic AAV delivery at birth, which instead induced CNM-like defects in muscle, underscoring the narrow therapeutic window, in terms of dose and developmental stage, for DNM2 modulation.

## Data Availability

The original contributions presented in this study are included in the article/Supplementary Material. Further inquiries can be directed to the corresponding author.

## Supplementary Materials

The following supporting information can be downloaded at: https://www.mdpi.com/article/10.3390/ijms27031471/s1.

## Abbreviations

The following abbreviations are used in this manuscript:

AAV	Adeno-associated virus
CMT	Charcot–Marie–Tooth
CNM	Centronuclear myopathy
ECM	Extracellular matrix
HE	Hematoxylin-eosin
SDH	Succinate dehydrogenase
SM	Striated muscles
TA	Tibialis anterior
Tg	Transgenic
Ub	Ubiquitous
WT	Wild-type
