# Supplementary material for: Muscle-Specific DNM2 Overexpression Improves Charcot–Marie–Tooth Disease In Vivo and Reveals a Narrow Therapeutic Window in Skeletal Muscle
Source: Int J Mol Sci. 2026 Feb 2;27(3):1471. doi: 10.3390/ijms27031471 (PMC12898409; doi:10.3390/ijms27031471)
Supplement: Supplementary file 1 [file ijms-27-01471-s001.zip › Supplementary Figures S1-S5 and Table S1.pdf]

Muscle-Specific DNM2 Overexpression Improves Charcot–Marie–Tooth Disease In Vivo and Reveals a Narrow Therapeutic Window in Skeletal Muscle

Marie Goret, Gwenaelle Piccolo and Jocelyn Laporte*

SUPPLEMENTARY MATERIALS

Supplementary Fig. S1 to S5.

Supplementary Table S1. Reagents used. Antibodies used for immunofluorescence and western blots. Primers used for PCR and qRT-PCR.

Supplementary Table S2. Number of mice used per test and statistical analysis performed. For each test, number total of mice and sex-repartition as well as statistical analysis.

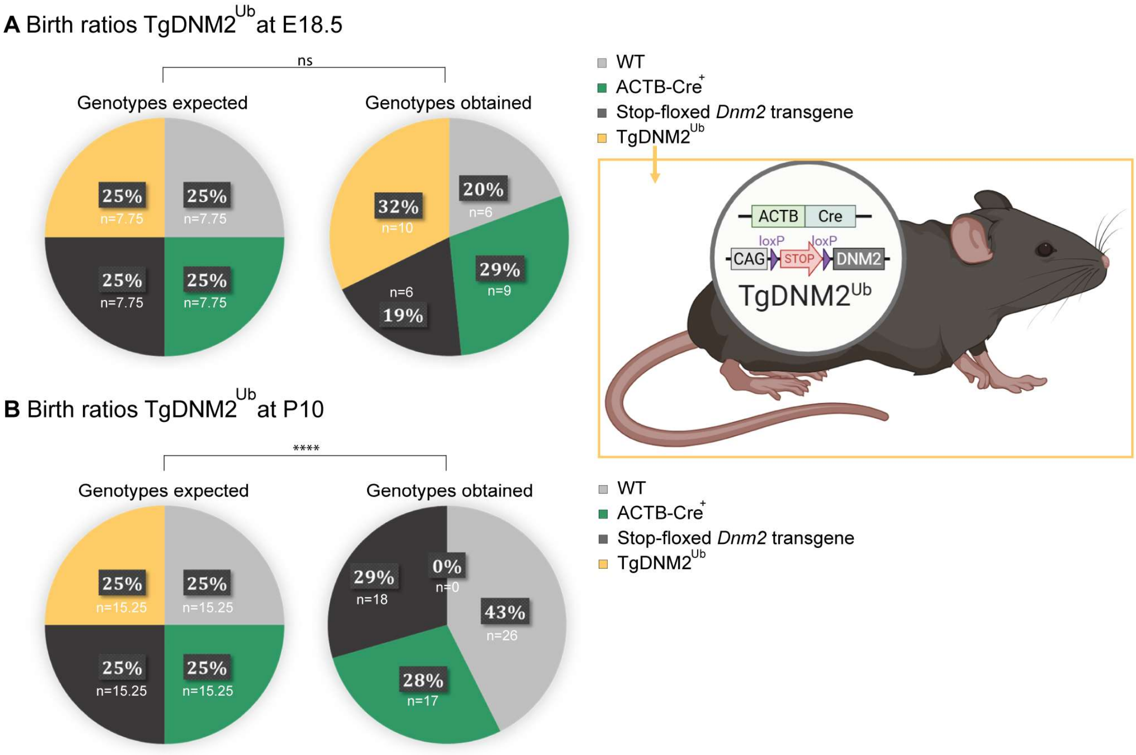

Group Name	Description
**WT**	No Cre, no *Dnm2* transgene
**ACTB-Cre^{+}**	Cre only
**Stop-floxed *Dnm2* transgene**	CAG-LoxP-STOP-LoxP-*Dnm2*
**TgDNM2^{Ub}**	Cre excises STOP → DNM2 overexpression

Supplementary Fig S1. The TgDNM2^{Ub} mouse line shows perinatal lethality. (A) Birth ratio expected and obtained at E18.5 from four different litters, in percentage and n number (*n*=31). **(B)** Birth ratio expected and obtained at P10 for the four groups in percentage and n number (*n*=61). ****p<0.0001. (A-B) Chi-squared test.

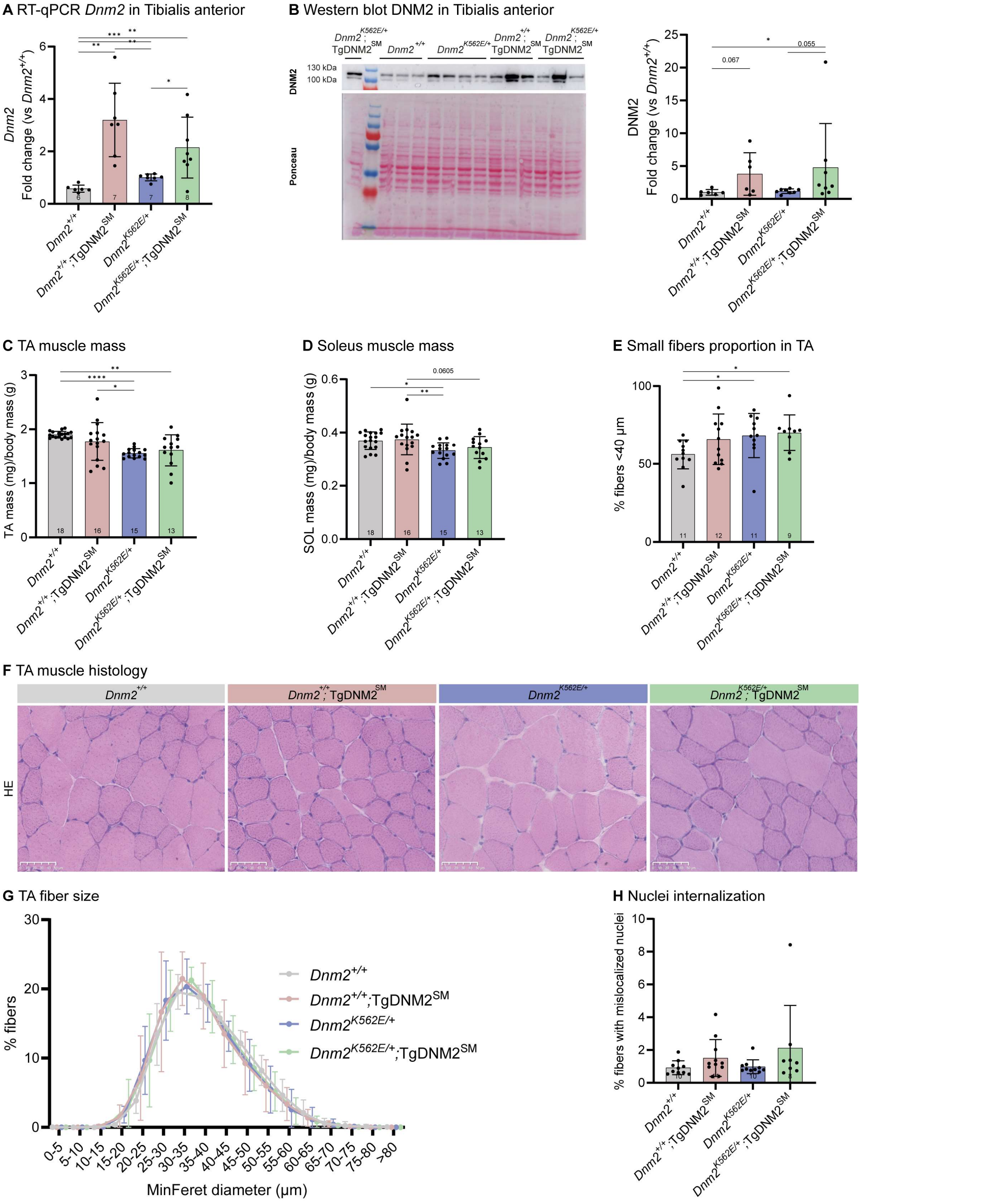

Supplementary Fig S2. Muscle-specific DNM2 overexpression does not improve *Dnm2*-CMT muscle atrophy. (A) RT-qPCR analysis of *Dnm2* expression in TA at 8w (6≤*n*≤8). **B)** Representative western blot and quantification of DNM2 protein in TA, normalized to Ponceau S staining (6≤*n*≤8). **(C-D)** Muscle mass of **(C)** TA and **(D)** Soleus normalized to body mass at 8w (13≤*n*≤18). **(E)** Proportion of small fibers (MinFeret<40 µm) in TA sections (9≤*n*≤12). **(F)** TA transversal sections stained with hematoxylin-eosin (HE). Scale bar= 50 µm. **(G)** TA fibers distribution based on their MinFeret diameter (9≤*n*≤12). **(H)** Proportion of fibers with internalized nuclei (8≤*n*≤11). Each dot represents a mouse. Values are represented as mean ± SD, *p<0.05, **p<0.01, ***p<0.001, ****p<0.0001. (A-E) ANOVA test. (H) Kruskal-Wallis test.

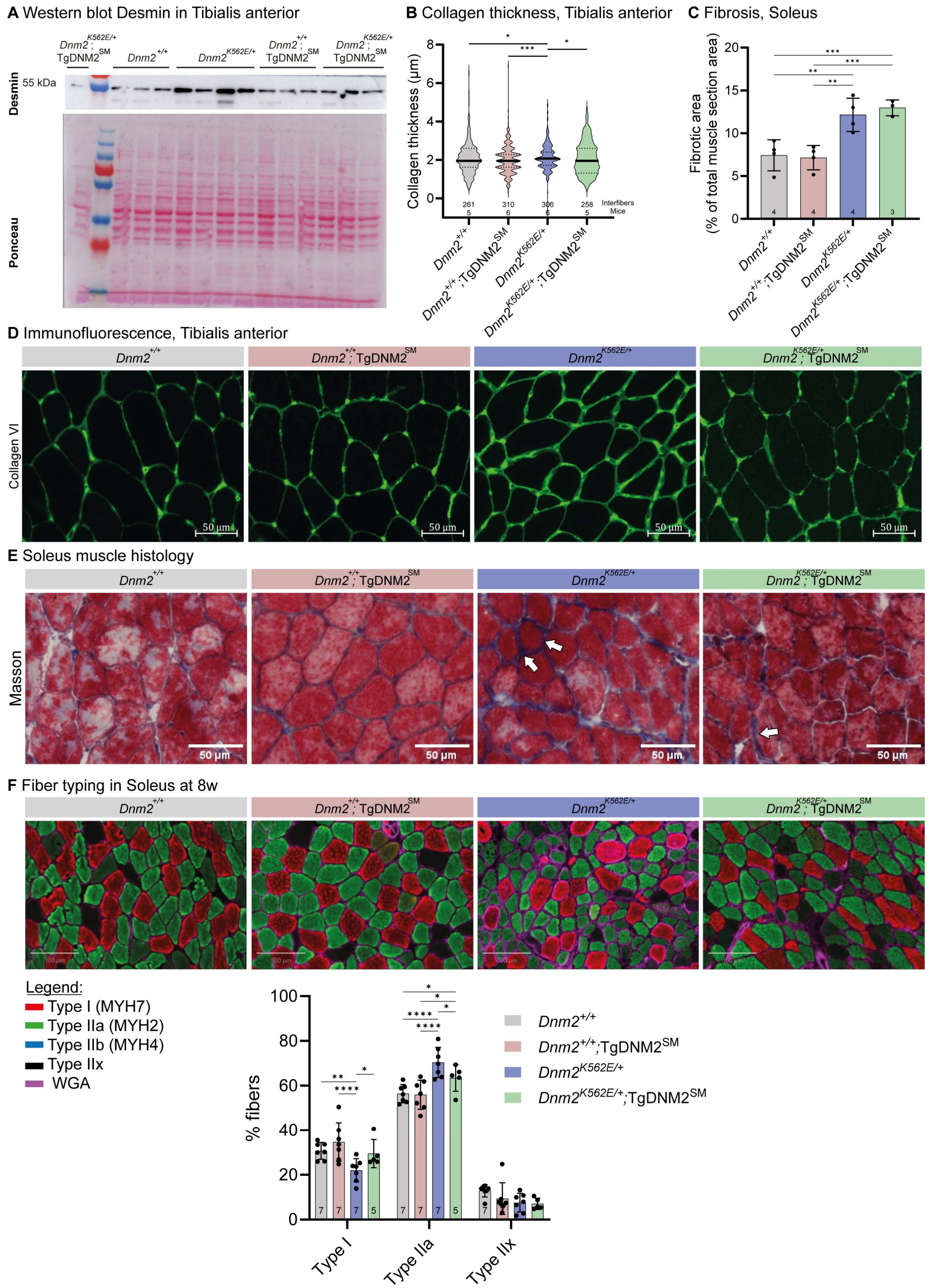

Supplementary Fig S3. Additional data on muscle-specific DNM2 overexpression from embryogenesis: key muscle protein expression, extracellular matrix and fiber type. (A) Representative western blot of Desmin protein level in TA at 8w and relative Ponceau S staining. **(B)** Collagen thickness (258≤*n*=interfibers≤310, 5≤*n*=mice≤6). **(C)** Fibrotic area in Soleus muscle (blue area on Masson's trichrome/total area) (3≤*n*≤4). **(D)** Immunolabelling of collagen VI in transversal TA sections. Scale bar= 50 µm. **(E)** Soleus transversal sections stained with Masson Trichrome; arrows indicate fibrosis. Scale bar= 50µm. **(F)** Immunolabelling of fiber types in transversal Soleus sections and quantification. Scale bar= 100 µm. (B) Individual interfiber spaces are plotted. (C, F) Each dot represents a mouse. Values are represented as mean ± SD, *p<0.05, **p<0.01, ***p<0.001. (B) Kruskal-Wallis test. (C, F) ANOVA test.

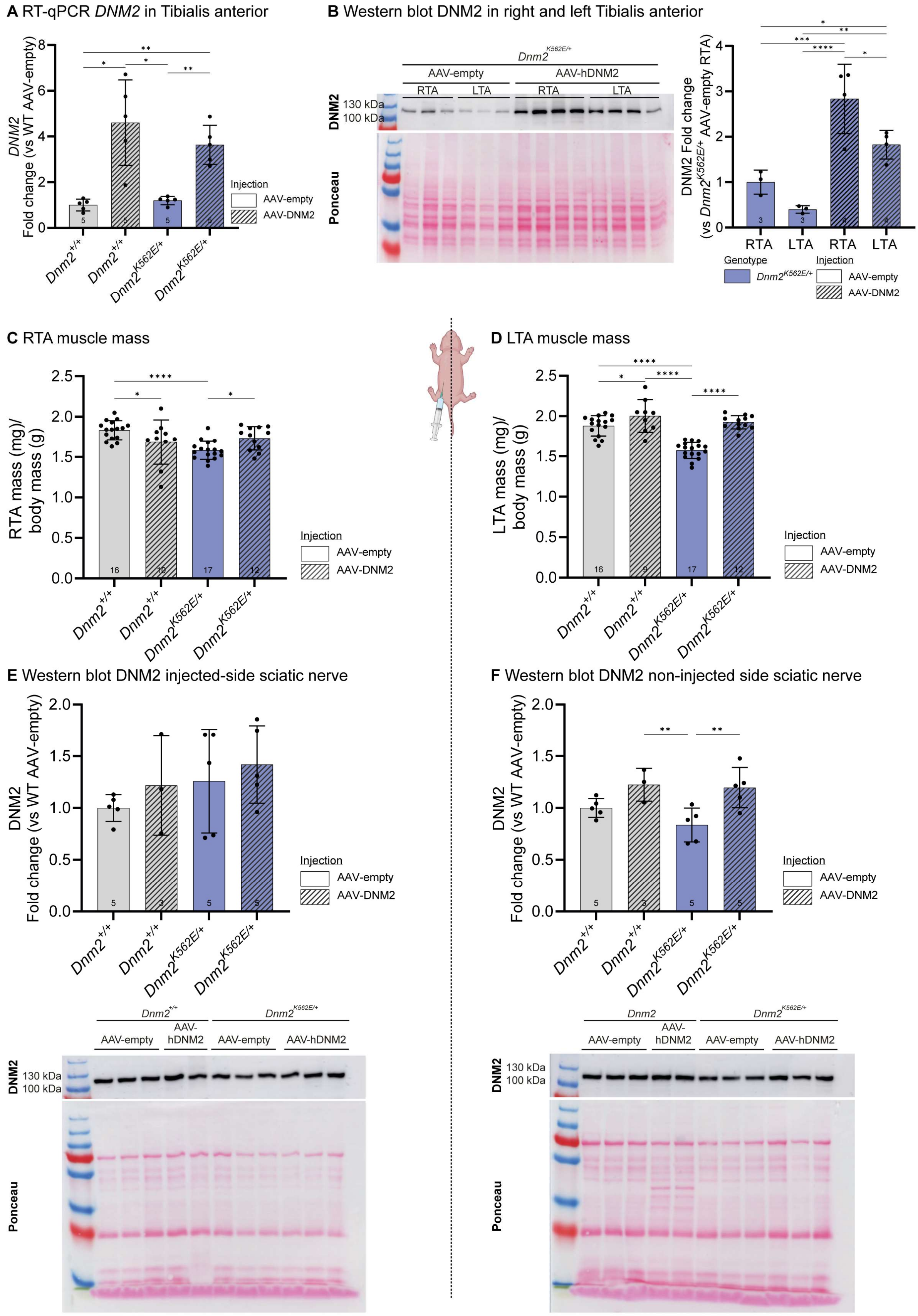

Supplementary Fig S4. Additional data on postnatal DM2 delivery for muscle and nerves. (A) RT-qPCR analysis of *Dnm2* expression in TA (random sides) at 8w (*n*=5). **(B)** Representative western blot and quantification of DNM2 protein level in right (RTA, IP-injected side) and left TA (LTA, contralateral side) in *Dnm2*$^{K562E/+}$ mice at 8w, normalized to Ponceau S staining (3≤*n*≤4). **(C-D)** TA muscle mass normalized to body mass at 8w: **(C)** RTA and **(D)** LTA (9≤*n*≤17). **(E-F)** Representative western blot and quantification of DNM2 protein level in sciatic nerve at 8w, normalized to Ponceau S staining in **(E)** IP-injected side (3≤*n*≤5) and **(F)** contralateral side (3≤*n*≤5). Each dot represents a mouse. Values are represented as mean ± SD, *p<0.05, **p<0.01, ***p<0.001, ****p<0.0001. (A-F) ANOVA test.

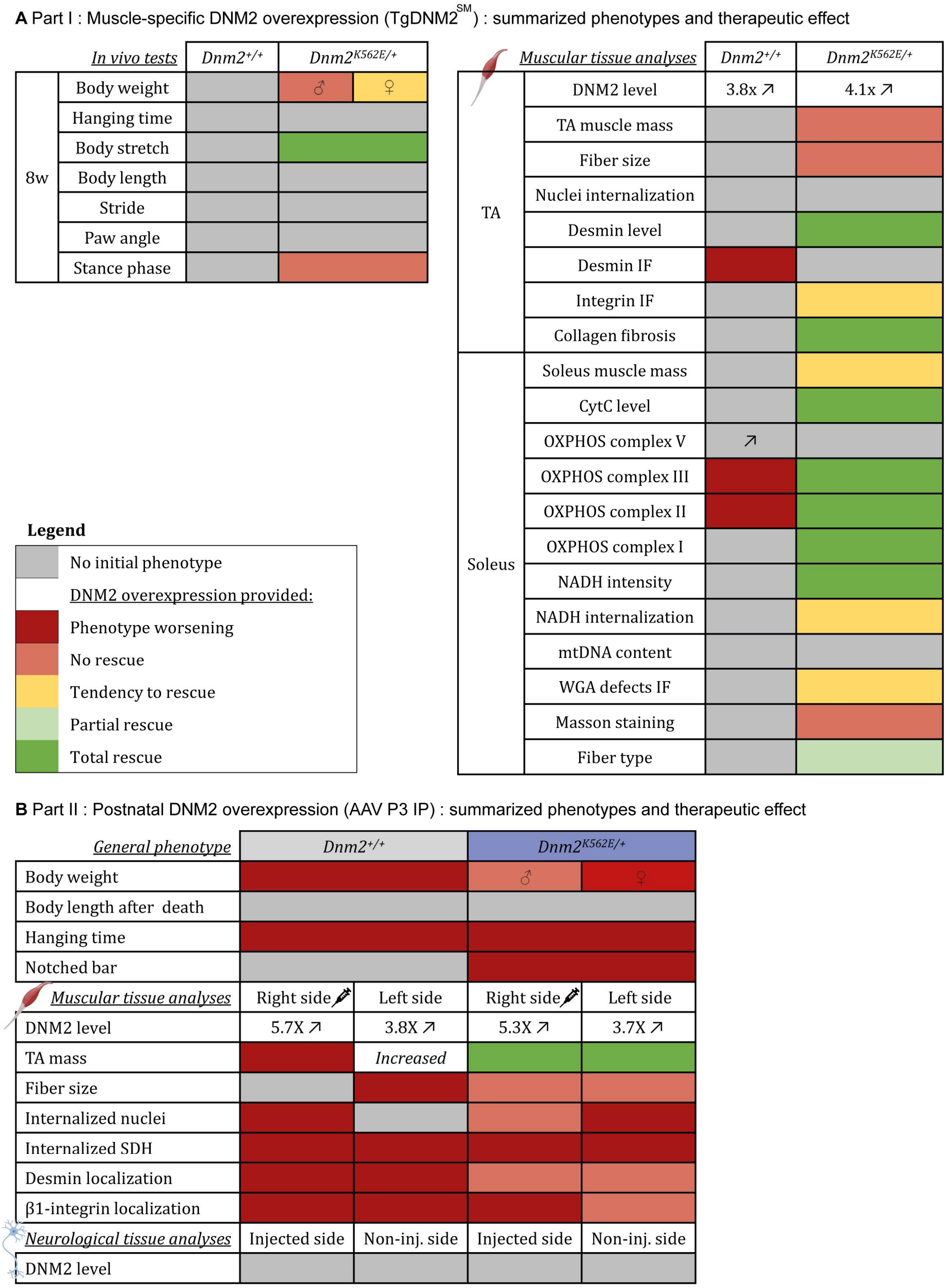

Supplementary Fig. S5. Overview of the improvements provided by DNM2 overexpression in *Dnm2$^{K562E/+}$* mice. (A) Summary of the therapeutic effects from transgenic overexpression of murine DNM2 in striated muscles of *Dnm2$^{+/+}$* and *Dnm2$^{K562E/+}$* mice from embryogenesis, based on in vivo tests and muscle tissue analyses at 8w. **(B)** Summary outcomes following intraperitoneal (IP) injection of human DNM2 at postnatal day 3 (P3) in control and *Dnm2$^{K562E/+}$* mice, with assessments at 8 weeks. TA= Tibialis anterior. Tendency to rescue= no significant difference between treated mutant mice and either untreated mutants or controls. Partial rescue= treated mutants differ from both untreated mutants and controls. Total rescue= treated mutants differ from untreated mutants but are similar to controls.

Reagent/Resource	Reference or Source	Identifier or Catalog Number
**Muscle immunofluorescence antibodies (dilution)**		
Integrin β1, rat monoclonal (1:250)	Sigma-Aldrich	MAB1997
↳ GARat Alexa 488, goat polyclonal (1:250)	Thermo Scientific	A-11006
Desmin, rabbit polyclonal (1:250)	Abcam	AB15200
↳ GAR Alexa 555, goat polyclonal (1:250)	Invitrogen	A21430
Collagen VI, rabbit polyclonal (1:250)	Novus Biologicals	NB 120-6588
↳ GAR Alexa 555, goat polyclonal (1:250)	Invitrogen	A21430
Anti-type I fibers: MYH7: mouse IG2b (1:50)	DSHB	BA-D5
↳ GAM IgG2b Cy3 (1:100)	Jackson Immunoresearch	115-165-207
Anti-type IIa fibers: MYH2: mouse IgG1 (1:50)	DSHB	SC-71
↳ GAM IgG1 Alexa 488 (1:100)	Jackson Immunoresearch	115-545-205
Anti-type IIb fibers: MYH4: mouse IgM (1:50)	DSHB	BF-F3
↳ GAM IgM DyLight 405 (1:100)	Jackson Immunoresearch	115-475-075
WGA, Wheat Germ Agglutinin, Alexa 647 (1:200)	Thermo Scientific	W32466
DAPI (1:1000)	/	/
**Western blot antibodies (dilution)**		
DNM2, rabbit polyclonal (1:1000)	Homemade (2865)	N/A
DNM2, rabbit polyclonal (1:1000)	ThermoFisher	PA5-19800
Desmin, rabbit polyclonal (1:1000)	Abcam	15200
OXPHOS, mouse monoclonal antibody cocktail (1: 1000)	ThermoFisher	45-8199
cytC, rabbit polyclonal (1:1000)	Cell Signaling	4272
GAR perox, goat polyclonal (1:10000)	Jackson Immunoresearch	111-036-045
GAM perox, goat polyclonal (1:10000)	Jackson Immunoresearch	115-036-068

**Genotyping PCR oligos**	5’-sequence-3’
6115 Er KE	TACACTGTCTGCACTGTCGAGCCCTG
6116 Ef KE	GCCATCTTCAACACAGAGCAGAGGTG
Cre 160	GAACCTGATGGACATGTTCAGG
Cre 161	AGTGCGTTCGAACGCTAGAGCCTGT
Sf 10692	GGCCCACCATTATCCGCCC
Wr 4035	CCTTTAAGCCTGCCCAGAAG
**AAV titration qPCR oligos**	
hDNM2 for	ATCAGGTGGACACTCTGGAGC
hDNM2 rev	GCATAGCTGATCTCCCGTCG
CMVe-CAG For5	TACGGTAAACTGCCCACTTG
CMVe-CAG Rev8	AGGAAAGTCCCATAAGGTCA
**qPCR oligos**	5’-sequence-3’
Rps11 for	CGCGTGGTGAATAAGGAAGC
Rps11 rev	GTAAGCACGCTCCGTCTGAA
Rpl27 for	AAGCCGTCATCGTGAAGAACA
Rpl27 rev	CTTGATCTTGGATCGCTTGGC
Dnm2 ex6	ACCCCACACTTGCAGAAAAC
Dnm2 ex8	CGCTTCTCAAAGTCCACTCC
Dnm2 ex10 m+h	GTCAAGCTGAAAGAGCCCTG
Dnm2 ex13 m+h	CTTCTTGTTCAGCTGCGTGC
Nd1 for	AAGTTGATCGTAACGGAAGC
Nd1 rev	CCCATTCGCGTTATTCTT

Supplementary Table S1. Reagents used. Antibodies used for immunofluorescence and western blots. Primers used for PCR and qRT-PCR.
