# Supplementary material for: Muscle-Specific DNM2 Overexpression Improves Charcot–Marie–Tooth Disease In Vivo and Reveals a Narrow Therapeutic Window in Skeletal Muscle
Source: Int J Mol Sci. 2026 Feb 2;27(3):1471. doi: 10.3390/ijms27031471 (PMC12898409; doi:10.3390/ijms27031471)

Supplementary material: Western blot
Unedited membranes

Fig. 2D/ Supplementary Fig. S3A
Desmin in TA

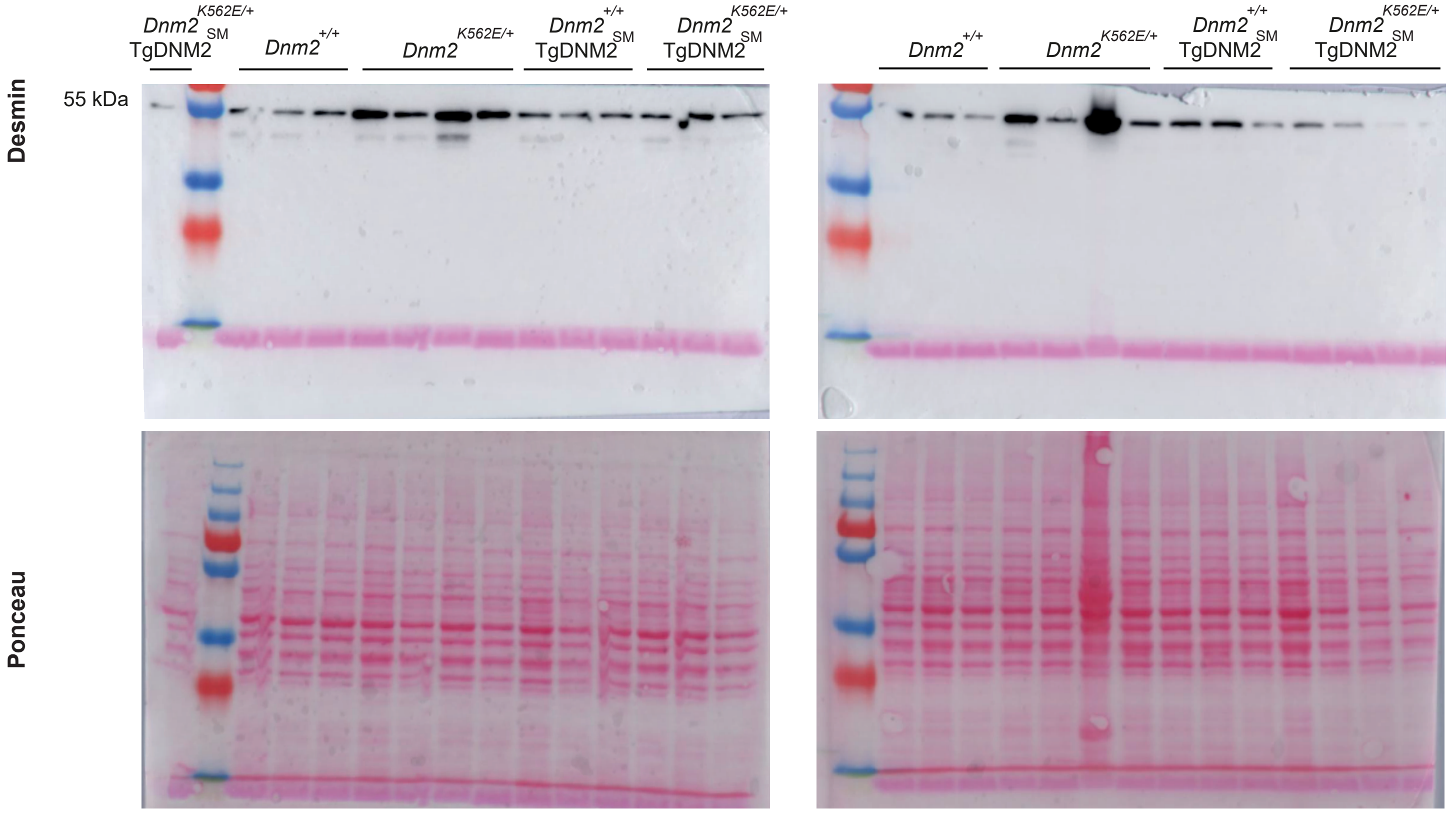

Fig. 3B
OXPHOS in Soleus

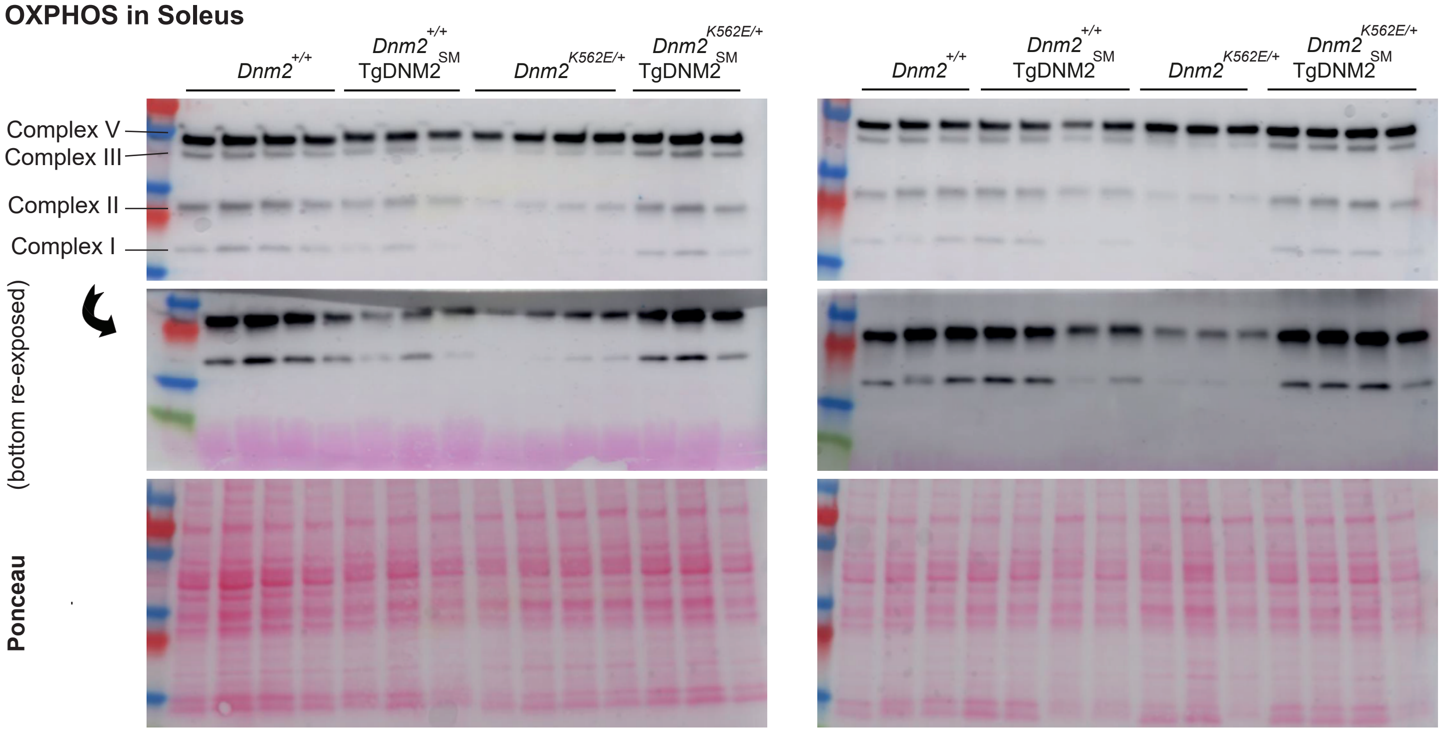

Fig. 3E
CytC in Soleus

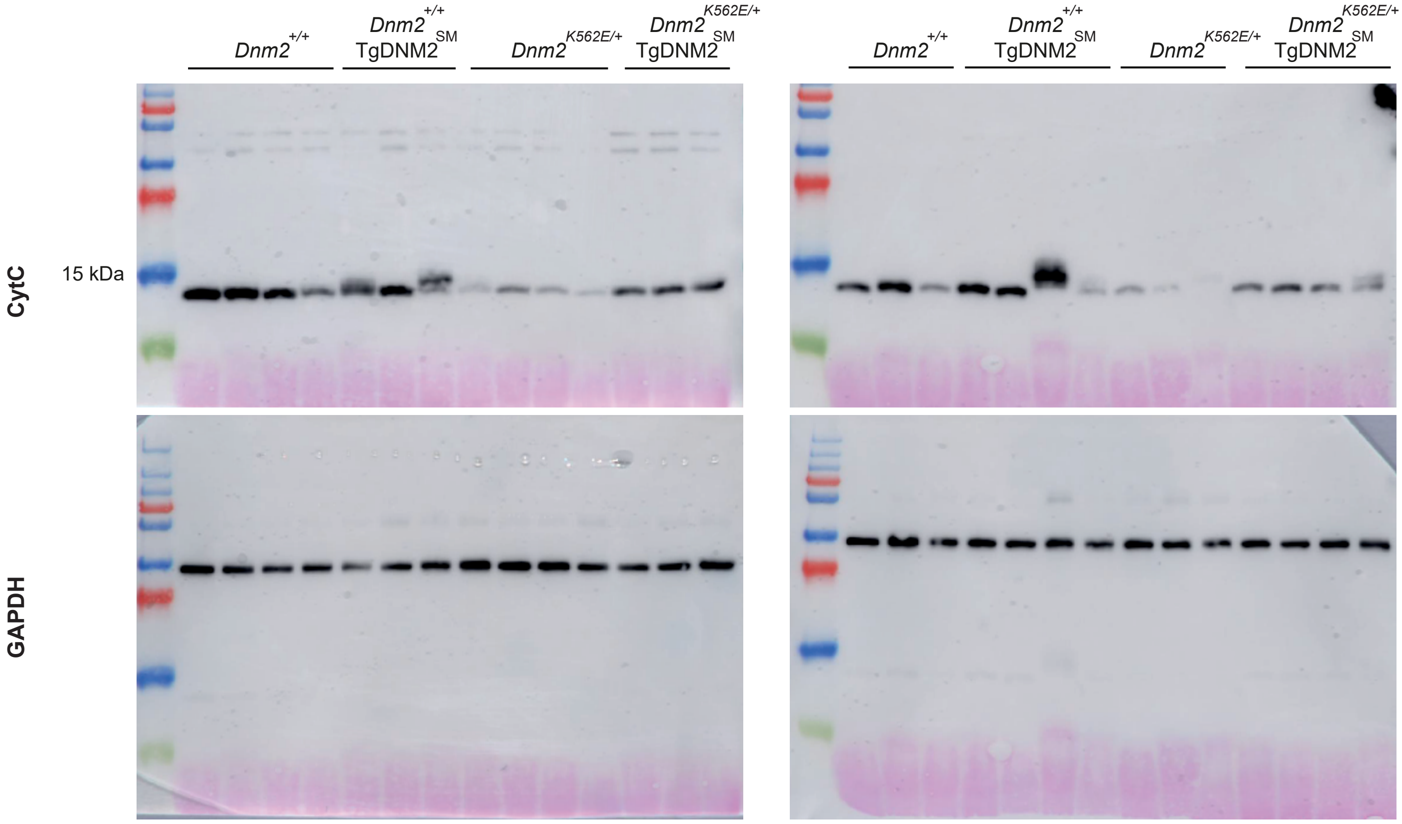

Fig. 4E
DNM2 in TA, right, injected side

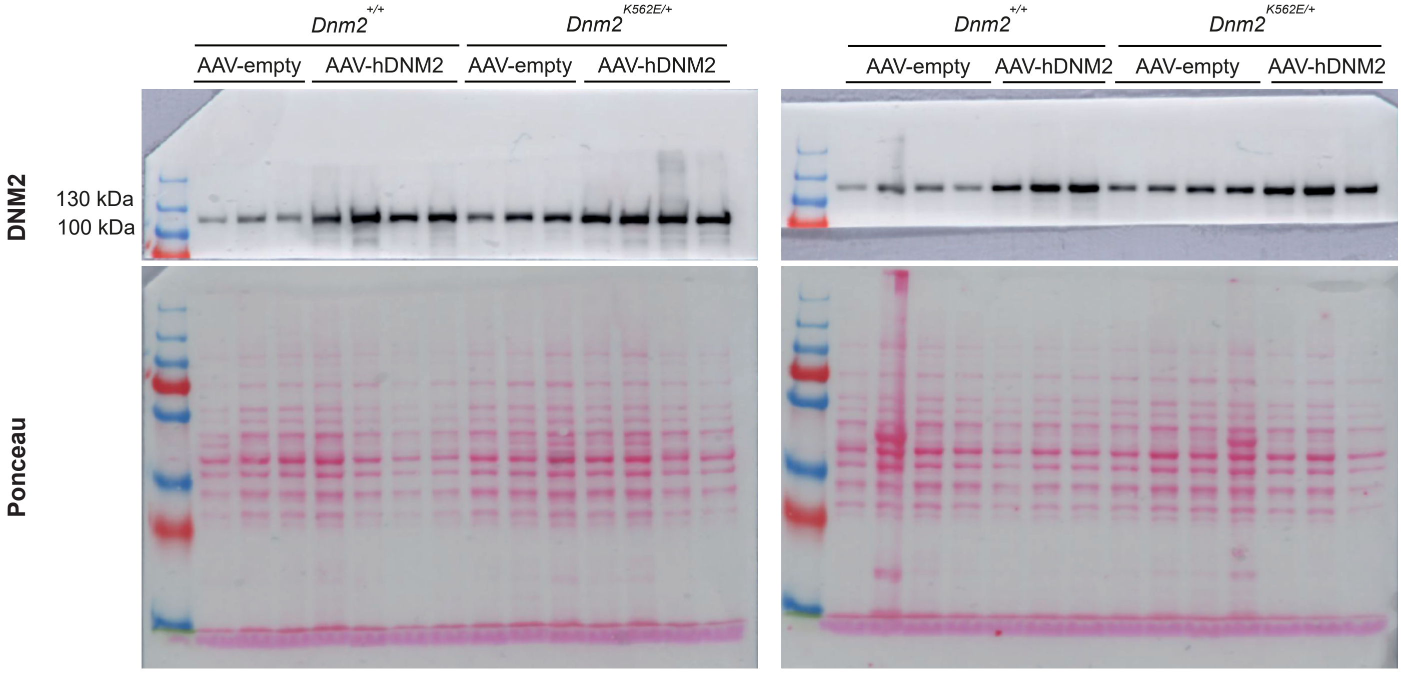

Fig. 4E
DNM2 in TA, left, opposite side

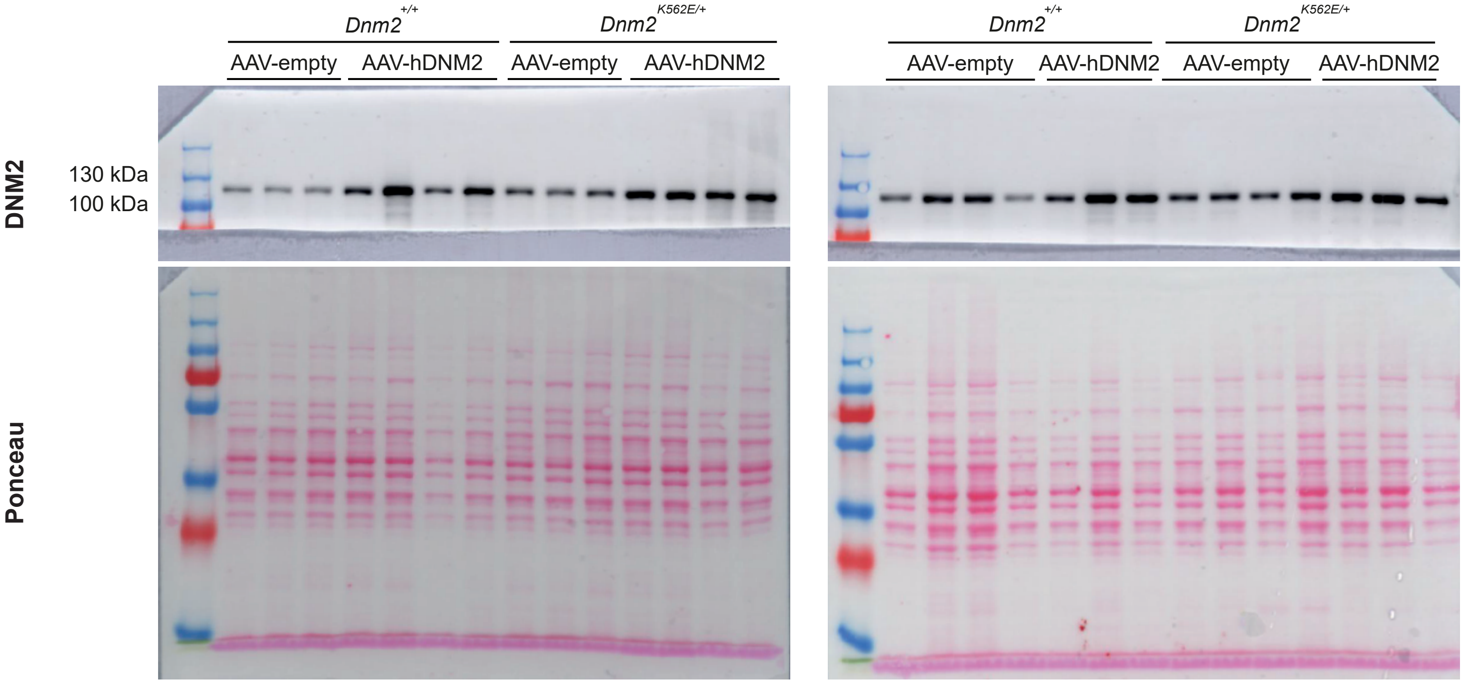

Supplementary Fig. S2B
DNM2 in TA

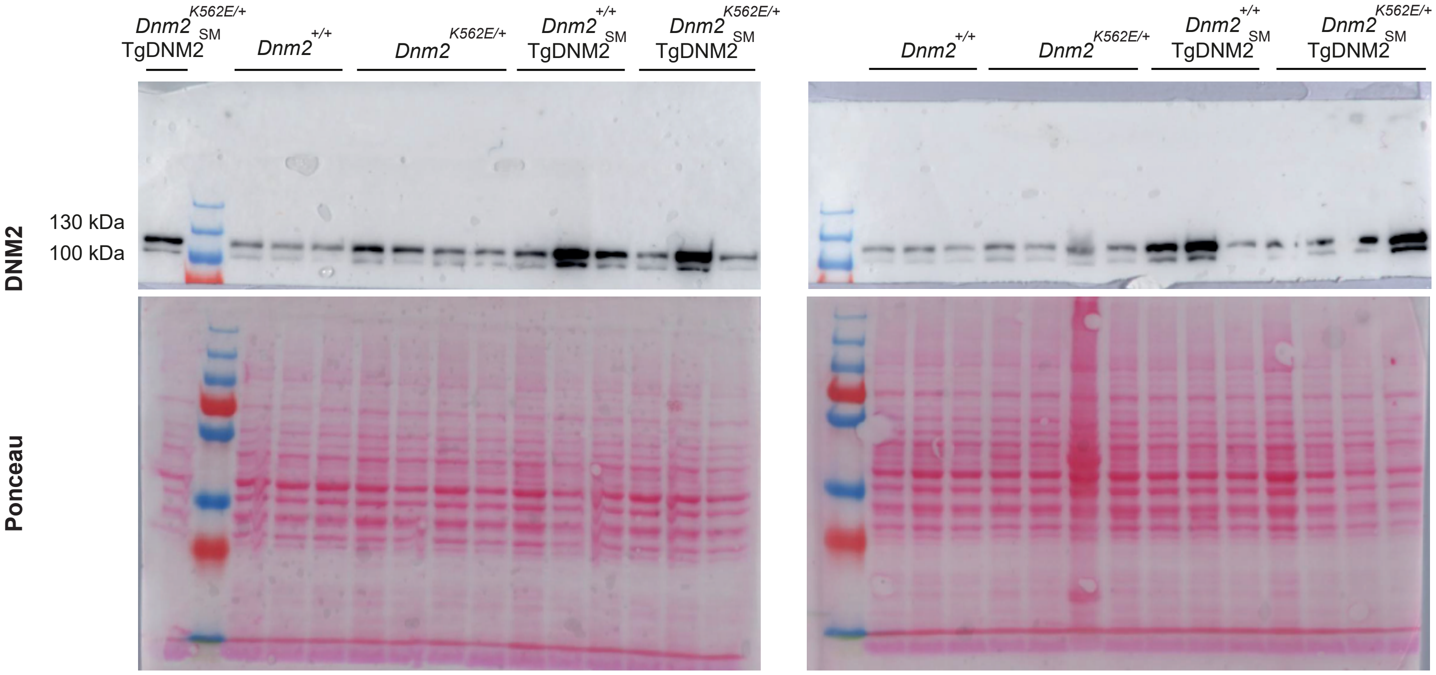

Supplementary Fig. S4B
DNM2 in TA, right injected side and left opposite side

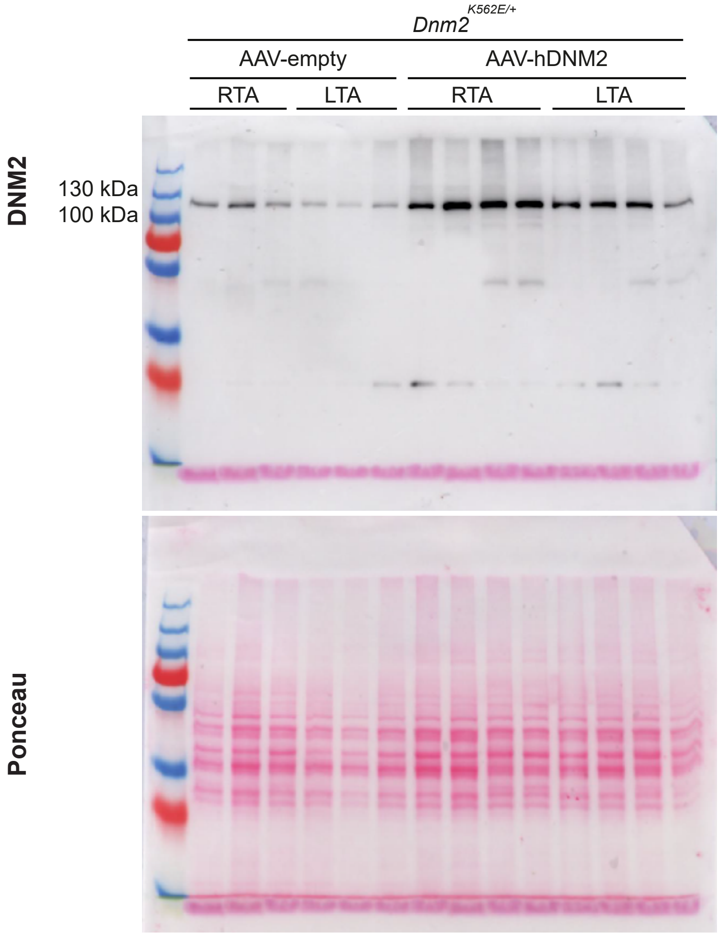

Supplementary Fig. S4E
DNM2 in sciatic nerve, injected side

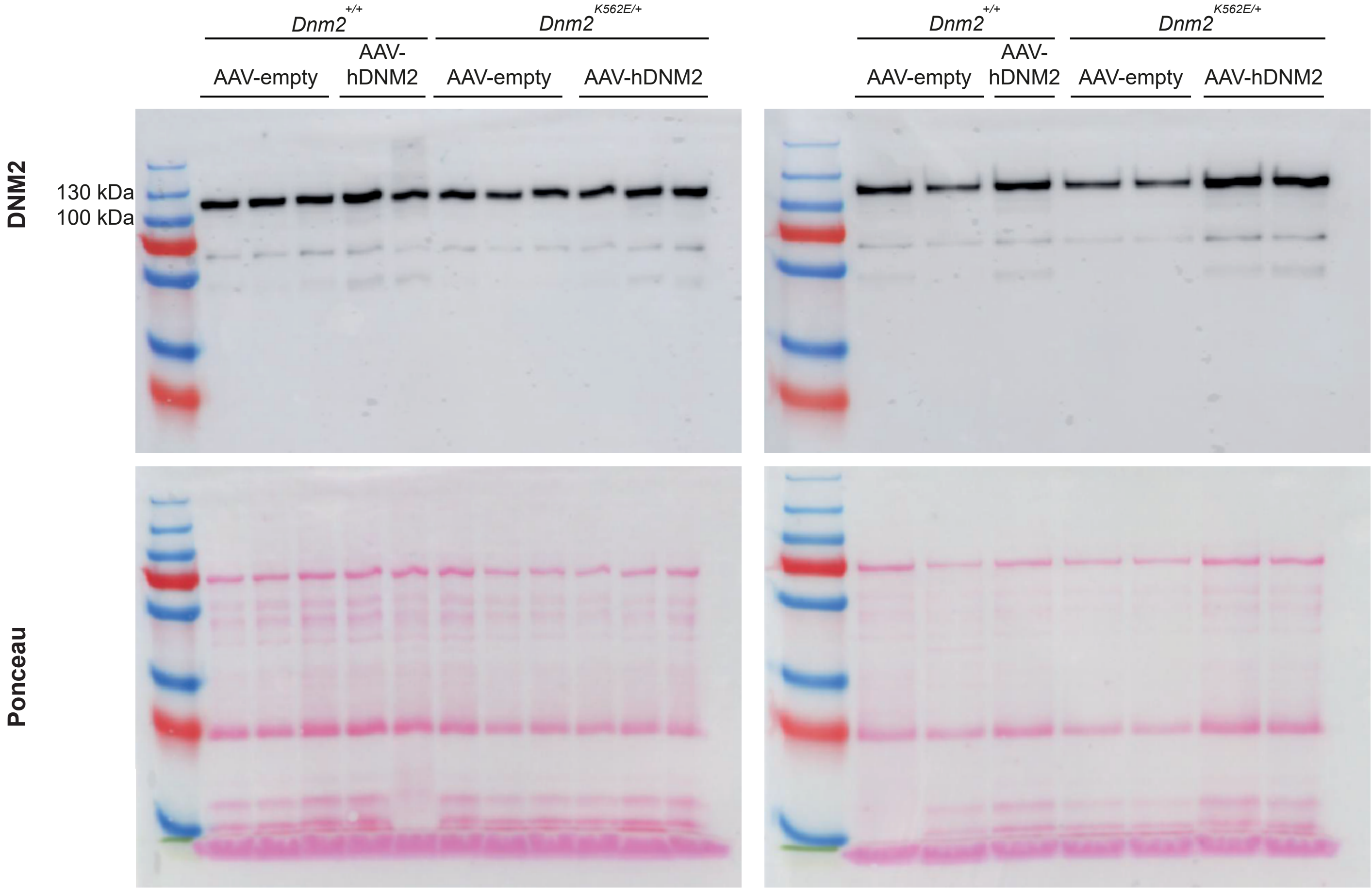

Supplementary Fig. S4F
DNM2 in sciatic nerve, opposite side

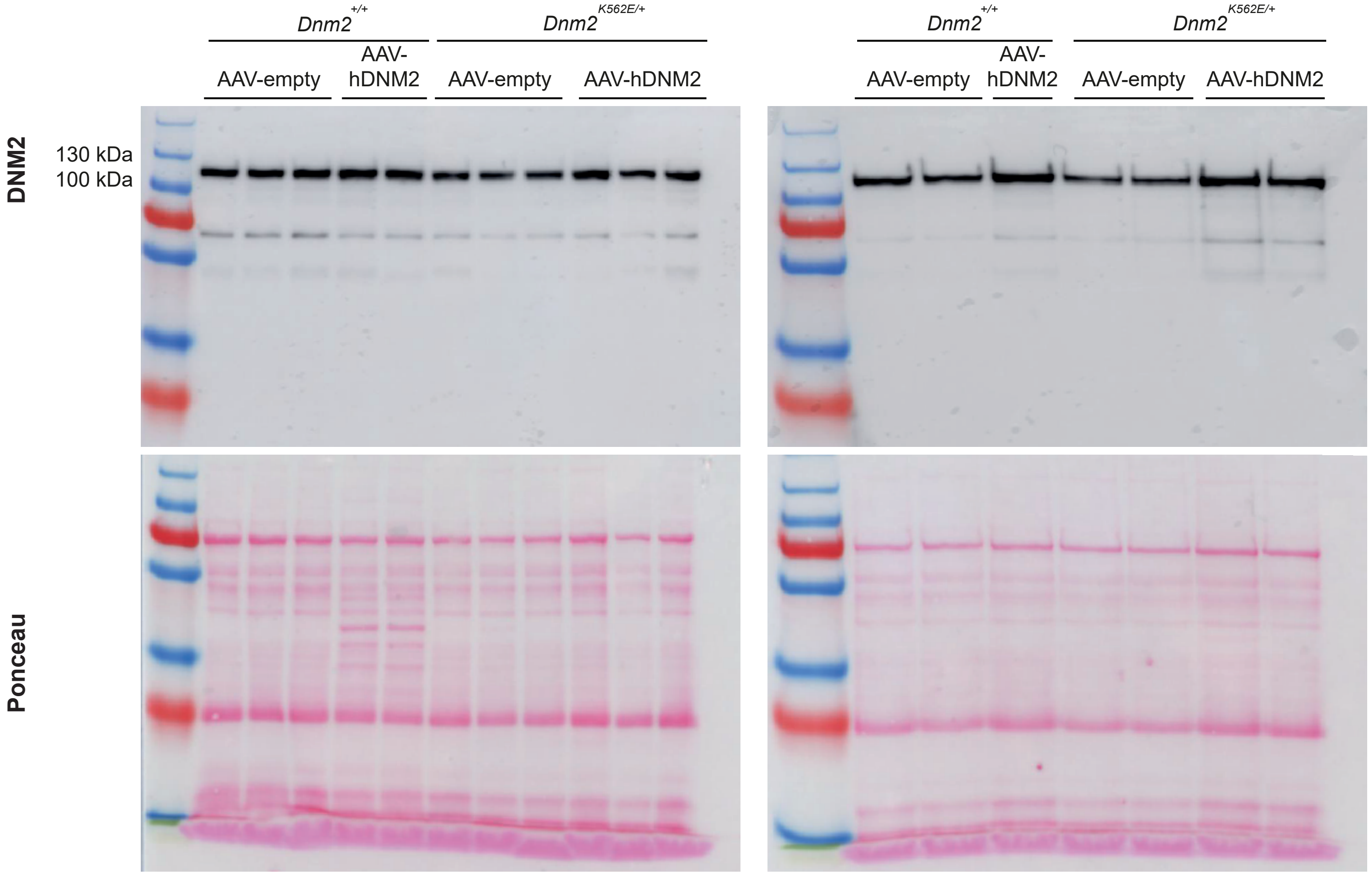

Supplement: Supplementary file 1 [file ijms-27-01471-s001.zip › Supplementary_Unedited_gels.pdf]
